# Nasal neuron PET imaging quantifies neuron generation and degeneration

**DOI:** 10.1172/JCI89162

**Published:** 2017-01-23

**Authors:** Genevieve C. Van de Bittner, Misha M. Riley, Luxiang Cao, Janina Ehses, Scott P. Herrick, Emily L. Ricq, Hsiao-Ying Wey, Michael J. O’Neill, Zeshan Ahmed, Tracey K. Murray, Jaclyn E. Smith, Changning Wang, Frederick A. Schroeder, Mark W. Albers, Jacob M. Hooker

**Affiliations:** 1 Athinoula A. Martinos Center for Biomedical Imaging, Department of Radiology, and; 2 Department of Neurology, Massachusetts General Hospital, Harvard Medical School, Charlestown, Massachusetts, USA.; 3 Department of Chemistry and Chemical Biology, Harvard University, Cambridge, Massachusetts, USA.; 4 Eli Lilly and Co. Ltd., Lilly Research Centre, Erl Wood Manor, Windlesham, Surrey, United Kingdom.

## Abstract

Olfactory dysfunction is broadly associated with neurodevelopmental and neurodegenerative diseases and predicts increased mortality rates in healthy individuals. Conventional measurements of olfactory health assess odor processing pathways within the brain and provide a limited understanding of primary odor detection. Quantification of the olfactory sensory neurons (OSNs), which detect odors within the nasal cavity, would provide insight into the etiology of olfactory dysfunction associated with disease and mortality. Notably, OSNs are continually replenished by adult neurogenesis in mammals, including humans, so OSN measurements are primed to provide specialized insights into neurological disease. Here, we have evaluated a PET radiotracer, [^11^C]GV1-57, that specifically binds mature OSNs and quantifies the mature OSN population in vivo. [^11^C]GV1-57 monitored native OSN population dynamics in rodents, detecting OSN generation during postnatal development and aging-associated neurodegeneration. [^11^C]GV1-57 additionally measured rates of neuron regeneration after acute injury and early-stage OSN deficits in a rodent tauopathy model of neurodegenerative disease. Preliminary assessment in nonhuman primates suggested maintained uptake and saturable binding of [^18^F]GV1-57 in primate nasal epithelium, supporting its translational potential. Future applications for GV1-57 include monitoring additional diseases or conditions associated with olfactory dysregulation, including cognitive decline, as well as monitoring effects of neuroregenerative or neuroprotective therapeutics.

## Introduction

Olfactory function may serve as a general marker of brain health. In neurodevelopmental disease, patients with intellectual disability, e.g., Down syndrome and idiopathic intellectual disability, or later-onset neurodevelopmental disorders, e.g., schizophrenia, show marked olfactory dysfunction (1–4). In the healthy aging population, olfactory dysfunction is correlated with cognitive decline (5, 6). Olfactory dysfunction is also a sign of neurodegenerative diseases, including the 2 most prevalent, Alzheimer’s disease (AD) and Parkinson’s disease (PD), as well as amyotrophic lateral sclerosis and Huntington’s disease (7, 8). In many AD and PD patients, hyposmia or anosmia, a partial or complete loss of the sense of smell, is detectable prior to cognitive decline in AD or motor dysfunction in PD (4, 9–13). Pilot studies have shown a high sensitivity for olfaction tests to predict conversion from mild cognitive impairment to AD (14, 15) with higher specificity than hippocampal volume measurements. Even after controlling for dementia, a recent study reveals that olfactory dysfunction in older adults predicts an increased mortality rate (16). Additionally, the increased mortality rates for adults with anosmia have been found to surpass the rates for adults with cancer or heart failure (17), with mortality consistently correlating with the severity of smell loss (16–18). Together, these studies indicate that olfactory health may be a broad marker for nervous system health.

The olfactory function measurement provided by smell identification tests used in the above-cited studies offers an incomplete picture of olfactory health. These functional odor identification tests survey higher-level olfactory processing, as opposed to the primary-level odor detection mediated by the olfactory sensory neurons (OSNs) within the superior nasal cavity. Biopsies of the OSN-containing nasal epithelium reveal loss of OSN density in patients with neurodevelopmental disease (Rett syndrome) as well as neurodegenerative disease (Alzheimer’s disease) (19, 20), suggesting that disease-related olfactory dysfunction extends to the primary olfactory pathway. In fact, the OSNs may be particularly vulnerable to neurological disease, since they are refreshed continually by adult neurogenesis in mammals, including humans (21–23). This property renders the OSNs a dynamic neuron population whose total neuron count is continuously dependent on the balance of neuron generation and neuron death, processes that are altered in neurological disease (24, 25). Direct quantification of OSNs would provide a measurement of olfactory health that is complementary to functional odor identification tests and would potentially provide an opportunity to monitor improved neural health through measurement of OSN regeneration.

For the measurement and quantification of OSNs, a noninvasive, whole-tissue analysis method would offer benefits over existing OSN analysis methods. The conventional histological analysis of nasal biopsies has revealed OSN losses in disease (19, 20); however, nasal biopsies are limited by their invasiveness, technical difficulty, and a significant sampling bias. Indeed, respiratory epithelial cells are often obtained instead of OSNs. Alternatively, noninvasive imaging techniques offer methods to assess the structural integrity and functional status of the olfactory neural system. Functional MRI (fMRI) studies have been used to assess changes in olfactory-related processing centers in AD patients (26–29). However, the OSNs provide a unique challenge to MRI techniques, particularly fMRI, due to the tissue thinness of the nasal epithelium and the surrounding air pockets. Noninvasive PET imaging of the OSNs using a radiotracer with high biological target specificity could surpass the limitations of both fMRI and nasal biopsies by providing a quantitative whole-tissue analysis.

To meet this need, we present a novel PET radiotracer, [^11^C]GV1-57, which enables objective, global measurement of the mature OSN population as well as alterations of OSN population through longitudinal imaging. [^11^C]GV1-57 was discovered during exploration of the structure-uptake relationships of several purine-based radiotracers. Unique to [^11^C]GV1-57 were a remarkably high signal-to-background and saturable binding within the nasal cavity. Determination of the cellular target of [^11^C]GV1-57, mature OSNs, was achieved by imaging targeted mature OSN death resulting from olfactory bulbectomy. Following this in vivo characterization, [^11^C]GV1-57 was applied to monitoring the OSN population across lifespan, which revealed an increasing OSN population to middle age, followed by OSN losses during aging. These data verify sensitivity to OSN population flux, the time-dependent change in the level of a neuron population. Assessment of OSN population fluctuations in individual animals during developmental periods indicated differences in individual rates of OSN influx, pointing to innate differences in OSN generation and degeneration rates.

In addition to monitoring normative alterations in OSN population, [^11^C]GV1-57 was applied to models of neuroregeneration and neurodegeneration. Following acute injury, longitudinal [^11^C]GV1-57 imaging uncovered increased rates of OSN influx that vary across individuals. This indicates that acute injury tunes OSN generation and death rates, which combine to regulate the mature OSN population, on an individual-dependent basis. To assess OSN population in a neurodegenerative disease that impacts olfactory function, [^11^C]GV1-57 was used in the rTg4510 tauopathy model of frontotemporal dementia/AD. In these animals, [^11^C]GV1-57 detected an OSN population deficit during the period of behavioral symptom onset, suggesting that the OSN population is impacted at early stages of neurodegenerative disease and OSN loss may contribute to the olfactory dysfunction measured early in AD progression.

Finally, the translational potential of GV1-57 was evaluated. Individual rodent GV1-57 PET images were assessed in a clinical radiology setting, indicating a high reliability for grouping WT and tauopathy animals. Additionally, preliminary application to nonhuman primates (NHPs) suggests that the GV1-57 radiotracer maintains good uptake and saturable binding in the primate nasal epithelium. Taken together, these results indicate that GV1-57 is a quantitative tool for measurement of the OSN population that provides insight into fluctuations in OSN population resulting from normative development, acute injury, neuron recovery, aging, and disease.

## Results

### Specific, saturable uptake of [^11^C]GV1-57 in the olfactory epithelium.

**Figure 1:**
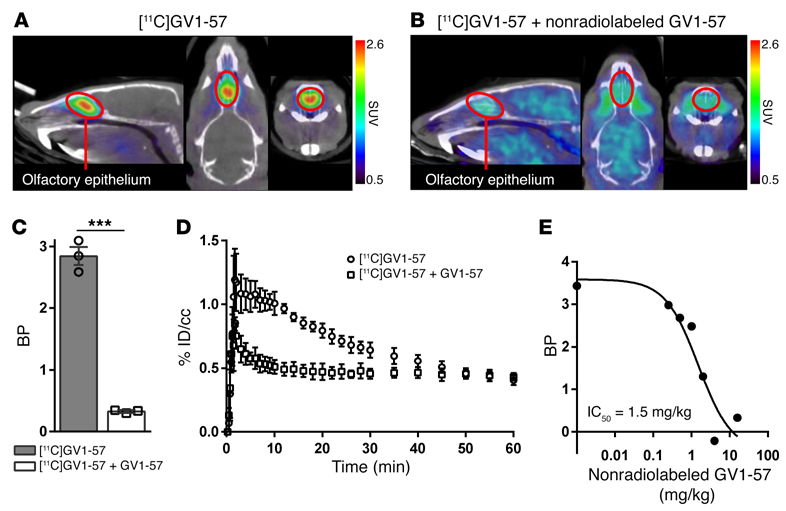
[^11^C]GV1-57 localizes to the OE and exhibits saturable binding. (**A**) Representative coregistered CT and [^11^C]GV1-57 PET images (SUV, NIH+white, 3–45 minutes) following treatment with [^11^C]GV1-57 (0.97 ± 0.097 mCi per animal). [^11^C]GV1-57 uptake is specific to the OE, which is circled in red in sagittal, transverse, and coronal views (left to right). (**B**) Representative [^11^C]GV1-57 PET images (SUV, NIH+white, 3–45 minutes) following pretreatment with nonradiolabeled GV1-57 (i.v., 16 mg/kg) 5 minutes prior to administration of [^11^C]GV1-57 (0.89 ± 0.032 mCi per animal). (**C**) Binding potential (BP) quantification of [^11^C]GV1-57 uptake in the OE of rats treated as described in **A** and **B**. BP was determined using a Logan analysis (Supplemental Figure 2). Error bars are ± SEM; *n* = 3 per group. ***P < 0.005 using a 2-tailed Student’s *t* test. (**D**) Averaged time-activity curves from rats treated as described in **A** and **B**. Error bars are ± SEM; *n* = 3 per group. %ID/cc, percent injected dose per cubic centimeter. (**E**) In vivo IC_50_ curve for [^11^C]GV1-57 (IC_50_ = 1.5 mg/kg, ~20 μM); rats coadministered with [^11^C]GV1-57 (0.52 ± 0.025 mCi) and increasing doses of nonradiolabeled GV1-57 (i.v., 0–4 mg/kg). Graph includes the [^11^C]GV1-57 BP obtained in **C** for animals pretreated with 16 mg/kg nonradiolabeled GV1-57.

[^11^C]GV1-57 contains a purinergic core minimally substituted with benzylamine, fluorine, and an isotopically interchangeable methyl group (Supplemental Figure 1; supplemental material available online with this article; https://doi.org/10.1172/JCI89162DS1). Although purinergic scaffolds are considered promiscuous because of their structural similarity to ATP, [^11^C]GV1-57 uniquely localizes to the olfactory epithelium (OE), as demonstrated by standardized uptake value (SUV) images in rodents (Figure 1A). [^11^C]GV1-57 demonstrates saturable binding with its specific biological target(s), as evaluated by pretreatment with the nonradiolabeled GV1-57, which is reflected as an 89% reduction in binding via Logan analysis (Figure 1, B and C). Logan analysis was completed using the brain as the reference region. The result is a linear Logan plot for [^11^C]GV1-57 injected alone or following treatment with nonradiolabeled GV1-57 (Supplemental Figure 2). Functional and binding inhibition assays of nonradiolabeled GV1-57 with adenosine-utilizing enzymes, including a panel of 365 kinases, 4 adenosine receptors, and 5 purinergic receptors, have ruled out binding to common purinergic binders (Supplemental Tables 1 and 2; Supplemental Figures 3 and 4; and ref. 30); additional target identification experiments are under way.

[^11^C]GV1-57 time-activity curve analysis from scans completed in the presence or absence of nonradiolabeled GV1-57 reveals relatively fast pharmacokinetics with specific target engagement between approximately 3 and 45 minutes and a nonsaturable component throughout the scan interval (Figure 1D). The in vivo IC_50_ (1.5 mg/kg) for [^11^C]GV1-57 was measured by cotreatment with increasing masses (0.25–4 mg/kg) of nonradiolabeled GV1-57 (Figure 1E). Accounting for the percentage uptake of [^11^C]GV1-57 in the OE (~1% injected dose per cubic centimeter; Figure 1D) results in an approximate in vivo OE IC_50_ value of 20 μM. This volumetric measurement does not account for the bones and air pockets surrounding the OE tissue and thus overestimates the in vivo IC_50_. Ex vivo autoradiography analysis suggests tighter binding with an IC_50_ of 0.33 μM (Supplemental Figure 5). Together, the initial characterizations of [^11^C]GV1-57 reveal its high, OE-specific uptake, suitable pharmacokinetic properties, and self-saturability, a quality necessary for PET measurement of receptor occupancy, protein density, or cellular population (31).

### [^11^C]GV1-57 uptake is associated with the mature OSN population.

**Figure 2:**
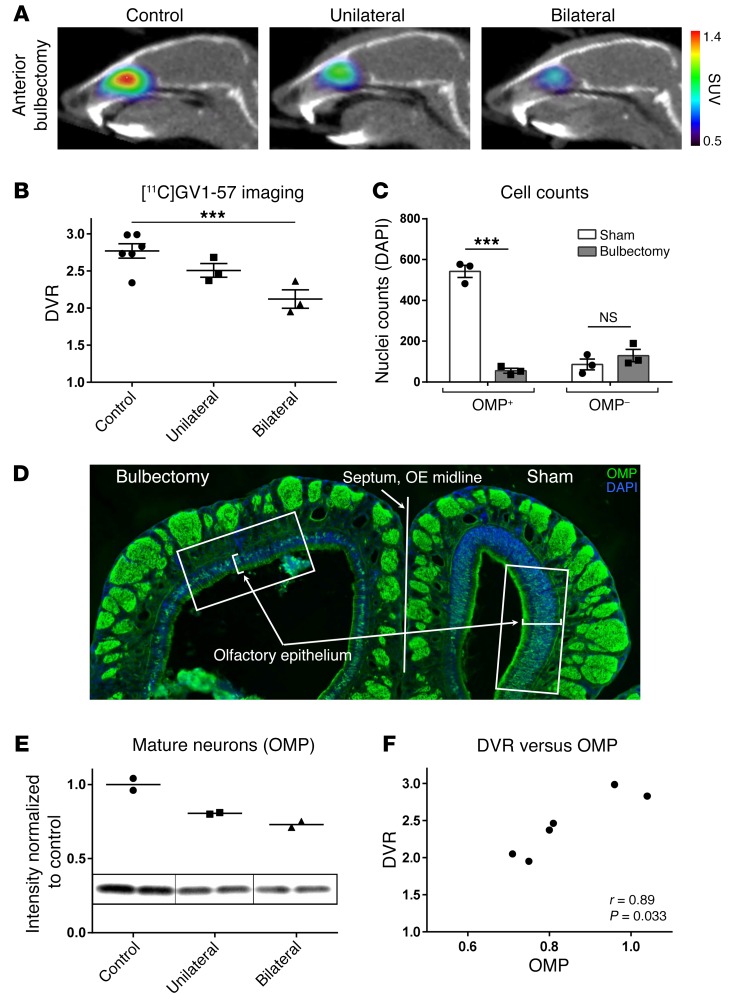
[^11^C]GV1-57 binds mature OSNs. (**A**) Representative [^11^C]GV1-57 PET images (SUV, NIH+white, 3–45 minutes) of 3-month-old mice imaged with no treatment (control) or 2–3 days after unilateral or bilateral anterior bulbectomy. (**B**) DVR quantification of [^11^C]GV1-57 uptake in control and bulbectomy-treated mice. Error bars are ± SEM; *n* = 3–6 per group. One-way ANOVA, *F*(2, 9) = 8.808, *P* < 0.01, followed by a 1-tailed Student’s *t* test, ***P < 0.005. (**C** and **D**) Coronal OE sections from a mouse that underwent unilateral anterior bulbectomy and [^11^C]GV1-57 imaging were immunostained with anti-OMP and stained with DAPI to provide total and OMP^+^ nuclei counts. OE regions with the greatest damage on the bulbectomy side were paired with the contralateral sham side, and both were counted. (**C**) OMP^+^ nuclei were significantly reduced following anterior bulbectomy, and OMP^–^ nuclei numbers were not significantly altered. Error bars are ± SEM; *n* = 3 tissue sections. ***P < 0.005 using a 2-tailed Student’s *t* test. NS, *P* = 0.34 using a 2-tailed Student’s *t* test. (**D**) Representative image of an immunostained OE section. White boxes indicate regions used for cell counting. (**E**) Western immunoblot of the mature OSN population (OMP) in control and bulbectomy-treated mice, using OE tissue from animals imaged with [^11^C]GV1-57. For this and the following immunoblot analyses, protein marker intensities relative to total protein loaded were used, since the goal was to analyze changes in relative cellular populations, as opposed to alterations in protein expression. *n* = 2 per group. (**F**) Significant correlation between [^11^C]GV1-57 quantification (DVR) and the mature OSN population (OMP) across individual mice (Spearman, *r* = 0.89, *P* = 0.033).

To identify the cellular localization of [^11^C]GV1-57 within the OE, we used a standard surgical rodent model for mature OSN-specific removal, olfactory bulbectomy (32, 33). This procedure cuts the mature OSN axons posterior to the cribriform plate, a partition that isolates the OE and mature OSN cell bodies from direct surgical or inflammatory damage. The mature OSNs with severed axons are selectively and robustly reduced 2–3 days after bulbectomy (32). To accomplish a graded mature OSN reduction, we completed unilateral and bilateral anterior bulbectomies. [^11^C]GV1-57 imaging 2–3 days after anterior bulbectomy resulted in a 15% decrease in average [^11^C]GV1-57 binding potential after unilateral bulbectomy and a 37% decrease [1-way ANOVA, *F*(2, 9) = 8.808, *P* < 0.01; post hoc 1-tailed *t* test, *P* < 0.005] following bilateral bulbectomy (Figure 2, A and B), indicating specificity for [^11^C]GV1-57 uptake within mature OSNs and sensitivity of [^11^C]GV1-57 to graded alterations in the mature OSN population.

The specificity of the anterior bulbectomy for mature OSN reduction was confirmed via cell counting of OE tissue sections immunostained for the mature OSN-specific olfactory marker protein (OMP) and the nucleus-specific DAPI (34). The OE from a unilaterally treated animal demonstrated markedly reduced OMP^+^ cell counts (*P* < 0.005) and stable OMP^–^ cell counts following bulbectomy (Figure 2, C and D), indicating selective loss of mature OSNs ipsilateral to the anterior bulbectomy. Cell counting was performed in a region prominently impacted by the bulbectomy to validate the specificity of the model for mature OSN reduction in an area with severe tissue loss. Notably, different regions throughout the OE show lesser or greater loss of mature OSNs (L. Cao, S.P. Herrick, M.W. Albers, unpublished observations). A whole-tissue measurement of OSN population, like the one provided by [^11^C]GV1-57 imaging, necessarily averages the mature OSN reductions over the whole tissue.

An assessment of the average mature OSN reduction in whole-tissue OE lysate following anterior bulbectomy was accomplished through Western immunoblotting. This analysis confirmed reduction of the mature OSN population, as assessed by probing for OMP (Figure 2E) (34). Comparison of the distribution volume ratio (DVR), a measure of [^11^C]GV1-57 binding, and OMP in individual mice across treatments indicates a significant correlation (Spearman, *r* = 0.89, *P* = 0.033; Figure 2F) between the 2 whole-tissue analysis methods, indicating that [^11^C]GV1-57 reliably reports on the mature OSN population.

Specific markers of other major OE cell types, immature OSNs (growth-associated protein 43, GAP43) and sustentacular cells (receptor accessory protein 6, REEP6) (Supplemental Figure 6 and refs. 35, 36), did not show a significant postbulbectomy correlation with [^11^C]GV1-57 binding by immunoblotting (Supplemental Figure 7), further supporting the association of [^11^C]GV1-57 with mature OSNs. In addition to confirming the [^11^C]GV1-57 cellular target, the bulbectomy model provides 1 mechanism for [^11^C]GV1-57 imaging of brain health — measurement of OSN loss resulting from CNS damage.

### Fluctuations in mature OSN population during postnatal development and aging.

**Figure 3:**
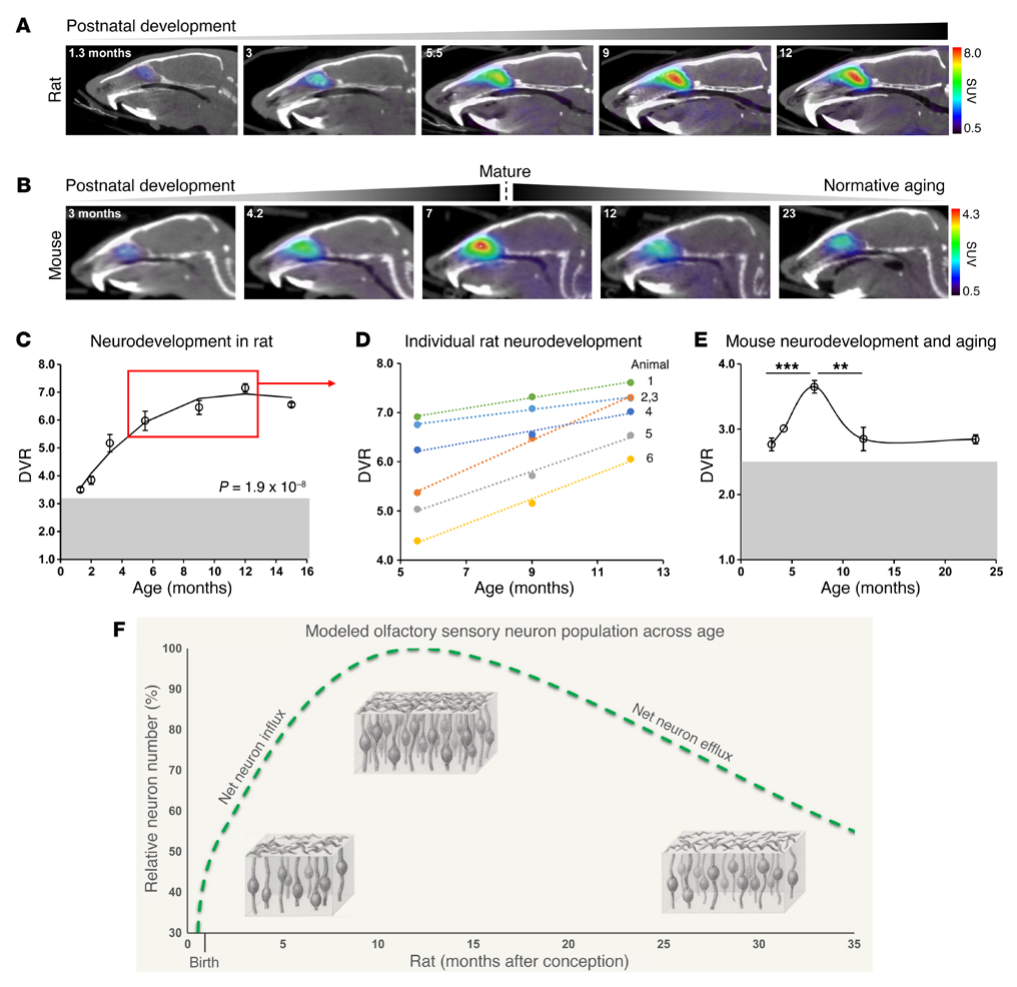
[^11^C]GV1-57 imaging of the mature OSN population during normative development and aging. (**A**) Representative [^11^C]GV1-57 PET images (SUV, NIH+white, 3–45 minutes) of male rats administered [^11^C]GV1-57 (0.93 ± 0.14 mCi) at select ages during postnatal olfactory neurodevelopment and into the OE developmental plateau. Images selected from 7 ages between 1.3 and 15 months old (**C**). (**B**) Representative [^11^C]GV1-57 PET images (SUV, NIH+white, 3–45 minutes) of female mice administered [^11^C]GV1-57 (0.54 ± 0.11 mCi) at select ages during postnatal olfactory neurodevelopment, maturation, and aging. (**C**) DVR quantification of [^11^C]GV1-57 uptake in rats imaged across OSN neurodevelopment and into the OE developmental plateau. Gray box indicates additional [^11^C]GV1-57 dynamic range. Error bars are ± SEM; *n* = 3–8 per age. Regression analysis indicates a cubic fit to the data; type III ANOVA with Satterthwaite approximation indicates significant curve fit, *P* = 1.9 × 10^–8^. (**D**) DVR quantification of [^11^C]GV1-57 uptake in individual rats imaged at 5.5, 9, and 12 months. The slope of each line indicates the rate of OSN influx for a single animal (Supplemental Table 3). (**E**) DVR quantification of [^11^C]GV1-57 uptake in mice imaged at 3, 4, 7, 12, and 23 months. Gray box indicates additional [^11^C]GV1-57 dynamic range. Error bars are ± SEM; *n* = 2–6 per age. **P < 0.01, ***P < 0.005, using a 2-tailed Student’s *t* test with Bonferroni correction (α = 0.025) for multiple comparisons. (**F**) Modeled olfactory neuron population curve using [^11^C]GV1-57 rodent imaging data from **C** and **E**. This model corroborates existing ex vivo OSN data (37–39) that suggest that net OSN influx occurs prior to a midlife OSN population peak with net OSN efflux beyond the peak. The mature OSN drawings depict changes in net OSN population, a result of changes to total OSN-populated area and OSN density across lifespan (37–39). Specifically, a less dense and smaller OE is present at early stages of development, a maximally dense and maximally large OE is present at the OSN population peak, and a less dense but still relatively large OE is present at older ages (37–39). Neuron illustrations were hand-drawn by GCV.

To assess the sensitivity of [^11^C]GV1-57 to the natural, longitudinal dynamics of the mature OSN population, we performed [^11^C]GV1-57 imaging at ages spanning normative neurodevelopment and aging-associated neurodegeneration. We imaged rats at 7 ages spanning 1.3 and 15 months (Figure 3A) and mice at 5 ages spanning 3 and 23 months (Figure 3B). A constant volume of interest (VOI) was used during image analysis to quantify OSN population, specifically OSN density within this region, across age. Between 1.3 and 15 months of age, we observed a statistically significant (*P* = 1.9 × 10^–8^) influence of age on [^11^C]GV1-57 binding in the rat OE with an apex, i.e., OSN population maximum, around 12 months of age (Figure 3C). Age-dependent imaging results were compared with OE cellular populations using extracts of dissected septal tissue from 2-, 3-, and 9-month-old rats. The OE tissue lysates were immunoblotted for specific markers of mature OSNs (olfactory marker protein, OMP), immature OSNs (GAP43), and sustentacular cells (REEP6); a discussion of these data can be found in Supplemental Figure 8.

Analysis of individual animals from 5.5 months to 12 months of age highlights individual rates of increased [^11^C]GV1-57 binding during postnatal development (Figure 3D). Here, the increase in [^11^C]GV1-57 signal signifies a net increase in the mature OSN population, which results from a net addition of neurons to the tissue, or net neuron influx. The rate of increase in [^11^C]GV1-57 binding, or rate of net mature OSN influx, varies across individual animals with a range of 0.084–0.30 DVR per month (Figure 3D and Supplemental Table 3). The individual variation in influx rates suggests that [^11^C]GV1-57 is monitoring individual physiological differences in OSN neurodevelopment rates, which represent a combination of neurogenesis, neuron maturation, and neuron death rates. Between 5.5 and 12 months of age, individuals starting with a DVR less than 5.5 exhibited a 2.4-fold higher mature OSN population growth rate (*P* < 0.005) compared with animals starting with a DVR greater than 6.0. This indicates that animals starting with a lower level of OSNs at specific developmental ages have subsequently increased neurogenesis and/or neuron maturation rates relative to neuron death rates when compared with animals starting with a higher mature OSN population.

The quantified [^11^C]GV1-57 signal was highly consistent in mice (Figure 3E), with increased binding during postnatal development into adulthood and a mature OSN population maximum around 7 months of age. Hereafter, reduced [^11^C]GV1-57 uptake is detected, validating [^11^C]GV1-57 sensitivity to aging-associated OSN neurodegeneration at relatively young ages and supporting the application of [^11^C]GV1-57 to neurodegenerative disease. Combined, the mouse and rat life-spanning imaging data support a mature OSN population model wherein total OSN numbers change across lifespan, resulting in a lifelong dynamic neuron population (Figure 3F). This mature OSN population model developed from the [^11^C]GV1-57 imaging data agrees well with existing articles, which combined suggest that there are an increasing OE tissue size and OSN density from birth into adulthood, followed by net OSN losses during aging (37–39). Thus, the unique power of noninvasive imaging has enabled a basic understanding of the dynamic nature of the mature OSN population and its fluctuations in individual animals. Future comparison of individual OSN population curves measured by [^11^C]GV1-57 with individual lifespan may provide insight into the relationship between homeostatic OSN population levels and aging and senescence, potentially providing a biomarker, i.e., mature OSN population, for health status during aging (17, 18).

### Visualizing neuron regeneration.

**Figure 4:**
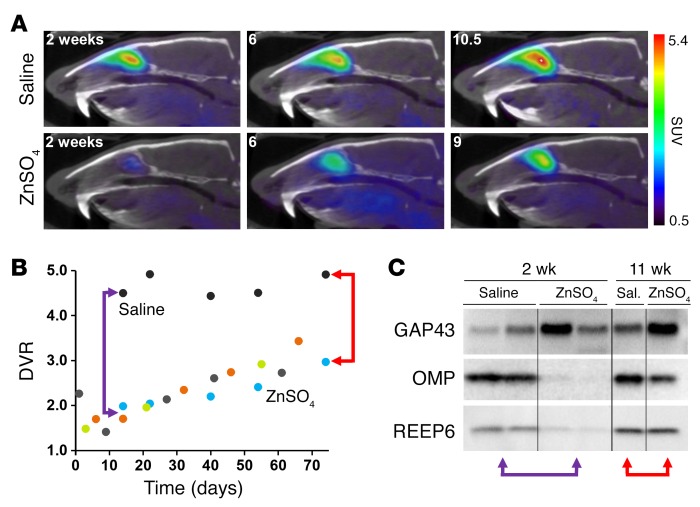
Longitudinal [^11^C]GV1-57 imaging monitors mature OSN regeneration. (**A**) Representative [^11^C]GV1-57 PET images (SUV, NIH+white, 3–45 minutes) of male rats treated intranasally with saline or ZnSO_4_ and imaged 0–10.5 weeks after treatment with [^11^C]GV1-57 (1.04 ± 0.19 mCi). All rats were approximately 3 months old when treated with saline or ZnSO_4_. (**B**) DVR quantification of [^11^C]GV1-57 uptake in saline control and ZnSO_4_-treated animals, each color indicating an individual animal monitored with [^11^C]GV1-57 over time. *n* = 1 saline-treated animal; *n* = 4 ZnSO_4_-treated animals. (**C**) Immunoblotting of immature neurons (GAP43), mature neurons (OMP), and sustentacular cells (REEP6) in septal OE tissue collected from animals 2 or 11 weeks after ZnSO_4_ or vehicle treatment. Red arrows indicate DVR values and the corresponding immunoblots of OE tissue collected from the same individual animals 11 weeks after treatment. Purple arrows indicate DVR values representative of [^11^C]GV1-57 imaging 2 weeks after treatment and corresponding immunoblots of OE tissue collected from a separate cohort of animals. *n* = 2 per group at 2 weeks after treatment; *n* = 1 per group at 11 weeks after treatment.

To longitudinally and noninvasively assess neurogenesis-stimulated mature OSN regeneration, we used an OE tissue ablation model. Intranasal irrigation with several chemical species results in complete or nearly complete removal of the major OE constituents (mature OSNs, immature OSNs, and sustentacular cells), subsequently inducing neurogenesis for mature OSN replacement (40–43). We intranasally administered a zinc sulfate (ZnSO_4_) solution to 4 Sprague-Dawley rats, and performed longitudinal imaging studies over 10.5 weeks (Figure 4A). Using the [^11^C]GV1-57 imaging data, we plotted a neuron population curve, which shows 2 phases: an initial phase of mature OSN death (net neuron efflux) and a subsequent phase of mature OSN regeneration (net neuron influx) (Figure 4B). The neuron death is relatively rapid, with a 64% reduction in [^11^C]GV1-57 binding after 18 hours and an additional 58% reduction over the next several days, affording a minimum OSN population after approximately 1 week. Beginning 2–3 weeks after treatment, we observed a net mature OSN influx at rates between 0.55 and 2.0 DVR/mo (Supplemental Table 4). As [^11^C]GV1-57 measures mature OSNs, the neural regeneration that is monitored relies on both neurogenesis and successful integration of the newly born neurons into the neural network.

A comparison with the normative OSN growth rate of 0.35 DVR/mo for rats between 3.2 and 5.5 months of age indicates that the OSN generation rate is higher for age-matched ZnSO_4_-treated animals (Supplemental Table 4), suggesting a higher rate of OSN neurogenesis and maturation following severe OSN loss. Additionally, the OSN influx rates after ZnSO_4_ exhibit a 3.7-fold difference between the lowest and highest individual OSN influx rates, demonstrating for a second time the sensitivity of [^11^C]GV1-57 to individual nuances in neuron population flux. These differences may stem from injury severity and/or individual variation in neurogenesis, neuron maturation, and neuron death rates, and future studies will be required to parse out the individual factors and their impact. On the whole, the group-level OSN efflux and influx timelines are in agreement with existing ZnSO_4_ literature (40–42); however, an individual rate analysis was previously unfeasible because of a lack of longitudinal OSN population monitoring tools.

For statistical analysis of the OSN population alteration after saline vehicle and ZnSO_4_ treatments, we imaged an additional cohort of untreated controls and saline- and ZnSO_4_-treated animals 2 weeks after treatment. No significant change in [^11^C]GV1-57 binding was observed in the saline vehicle cohort relative to the untreated controls, but [^11^C]GV1-57 binding was diminished 85% in the ZnSO_4_ cohort (*P* < 0.005) (Supplemental Figure 9). Immunoblotting of OE extracts prepared from these animals 2 weeks after treatment confirmed a nearly complete loss of markers of mature OSNs and sustentacular cells, with an increase in the immature OSN marker, GAP43 (Figure 4C). Immunoblotting of OE tissue obtained from longitudinally imaged animals 11 weeks after treatment indicated a complete return of the sustentacular cells, a persistent increase of immature OSNs, and a 67% return of mature OSNs (Figure 4C and Supplemental Figure 10). This incomplete mature OSN recovery 11 weeks after ZnSO_4_ is consistent with the corresponding intraindividual [^11^C]GV1-57 imaging signature, which shows a 50% return in [^11^C]GV1-57 binding. Across bulbectomy, developmental, and ZnSO_4_ animal models, the data demonstrate a consistent correlation of [^11^C]GV1-57 binding and mature OSN levels, as assessed by OMP immunoblotting, that is not consistently found for the other major OE cellular constituents, the immature OSNs and the sustentacular cells.

The wide range of OSN populations present in the ZnSO_4_ model allowed comparison of DVR and SUV values across OSN population levels within an individual animal. Analysis of [^11^C]GV1-57 DVR versus SUV using data from a ZnSO_4_-treated animal indicates a significant correlation of the 2 measurements (Spearman, *r* = 1.0, *P* = 0.0028) (Supplemental Figure 11), further validating either measure for quantitative analysis of [^11^C]GV1-57 imaging across treatments, genetic models, or time.

### [^11^C]GV1-57 imaging reveals an olfactory neuron deficit in a tauopathy model.

**Figure 5:**
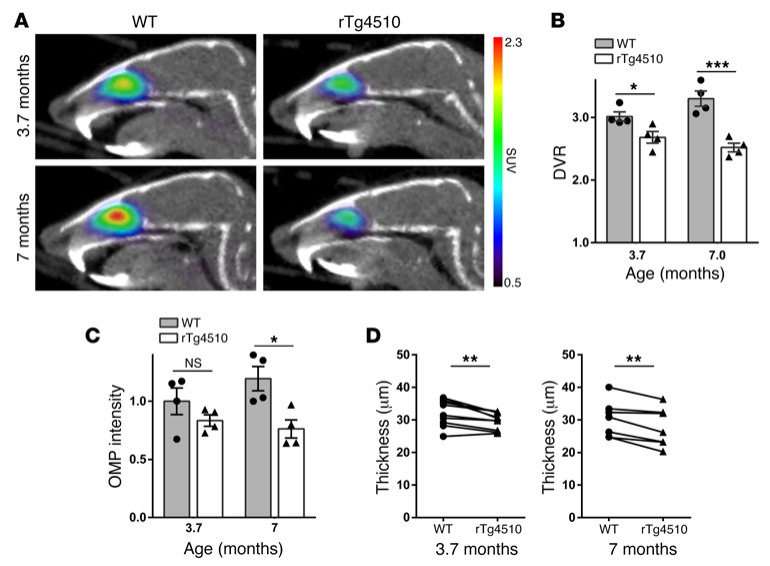
[^11^C]GV1-57 imaging of early-symptomatic mature OSN neurodegeneration in a murine tauopathy model. (**A**) Representative [^11^C]GV1-57 PET images (SUV, NIH+white, 3–45 minutes) of female rTg4510 and WT (within colony) mice at 3.7 and 7 months of age. Mice were administered [^11^C]GV1-57 (0.51 ± 0.091 mCi) and imaged for 60 minutes. (**B**) DVR quantification of [^11^C]GV1-57 uptake in WT control and rTg4510 animals. Error bars are ± SEM; *n* = 4 per group. *P < 0.05, ***P < 0.005 using a 2-tailed Student’s *t* test. (**C**) Immunoblot quantification of the mature OSN population (OMP) in WT and rTg4510 animals at 3.7 and 7 months of age. Immunoblot intensities were multiplied by total protein extracted per sample and normalized to the mean of the WT 3.7-month values to determine relative mature OSN populations. Error bars are ± SEM; *n* = 4 per group. *P < 0.05 using a 2-tailed Student’s *t* test. NS, *P* = 0.26 using a 2-tailed Student’s *t* test. (**D**) OE thickness measurements from paired (within age) septal OE regions. OE regions were paired by OE depth and OE subregion, i.e., the dorsal or medial portion of the septum. Error bars are ± SEM; *n* = 6–8 regions per group. **P < 0.01 using a 2-tailed, paired Student’s *t* test.

Emboldened by the [^11^C]GV1-57 detection of OSN regeneration and aging-associated neurodegeneration, we assessed the OSN population in a neurodegenerative model, the murine rTg4510 tauopathy model. This model overexpresses pathogenic tau protein in the hippocampus, cortex, and olfactory bulb (44) and develops a severe neurodegenerative phenotype, particularly in the adult neurogenic dentate gyrus (DG) and the frontal cortex (44, 45), with concomitant negative effects on memory and behavior. In the DG, neurodegeneration occurs months before tau deposition (45). Given that the OE and DG exhibit adult neuron turnover, we anticipated early neurodegenerative changes in the OE. [^11^C]GV1-57 imaging was performed in rTg4510 mice at 3.7 and 7 months of age (Figure 5A). DG neurodegeneration, but not cortical neurodegeneration, is seen at the younger age (3.7 months), whereas severe cortex neurodegeneration and widespread tau tangles are present at 7 months (44, 45). At both ages, significant reductions in [^11^C]GV1-57 binding, 17% (*P* < 0.05) at 3.7 months and 34% (*P* < 0.005) at 7 months, were found in the rTg4510 animals relative to age-matched controls (Figure 5B), indicating an OSN population deficit. We hypothesize translation of this early-stage OSN deficit to human tauopathies given the similar adult neurogenic nature of human OSNs (21–23) and existing biopsy data from Alzheimer’s patients (20). Verifying this novel neurodegenerative OSN phenotype was a reduced postmortem OMP staining (36% at 7 months, *P* < 0.05) (Figure 5C and Supplemental Figure 12) and OE thickness (8% at 3.7 months, *P* < 0.005; 9% at 7 months, *P* < 0.01) (Figure 5D), which has a linear relationship with neuron number (39). OMP levels were not significantly reduced at 3.7 months, although the percentage decrease from WT to rTg4510 animals (17%) was equivalent to that found with [^11^C]GV1-57 imaging, pointing to a lower variability for [^11^C]GV1-57 quantification.

The early-stage OSN neurodegeneration detected by [^11^C]GV1-57 was compared with tau tangle deposition and brain thickness of 2-, 4-, 6-, and 8-month-old rTg4510 animals (Supplemental Figure 13). Tau tangle pathology begins between 2 and 4 months of age, while reduced brain thickness occurs after [^11^C]GV1-57–detected OSN deficits, between 4 and 6 months of age. Thus, [^11^C]GV1-57 offers a marker of neurodegeneration relatively early during the progression of tauopathy in this transgenic model. Notably, neuron loss is a better correlate of cognitive deficits than tau burden within the same brain region (46, 47), and tau pathology can be present without neuron loss (48). Therefore, [^11^C]GV1-57 imaging of early-stage neuron loss in human neurodegenerative disease may offer complementary diagnostic and therapeutic monitoring applications compared with existing biomarkers.

### Translational potential: clinical image assessment and nonhuman primate imaging.

The clinical translational potential of [^11^C]GV1-57 was first evaluated using the WT and rTg4510 imaging data in a simulated clinical setting. Individual mouse scans were read by experienced radiologists not familiar with [^11^C]GV1-57 imaging. Following presentation of a training slide with representative WT and rTg4510 images, the radiologists were asked to use the scan data to classify individual animals by genotype. From the 3.7-month-old animals, 83% were grouped correctly with 1 false positive, and from the 7-month-old animals, 100% were grouped correctly (Supplemental Tables 5 and 6). The assessment of GV1-57 images in a clinical setting highlights GV1-57’s potential for clinical, image-based detection of OSN neurodegeneration, supporting the future application of [^11^C]GV1-57 to human imaging.

Interspecies differences are an important consideration for the translation of any radiotracer. The radiotracer target density is of paramount consideration, as alterations in the radiotracer target density (i.e., concentration) will impact radiotracer binding, and consequently radiotracer uptake. The density of the GV1-57 target, the mature OSNs, has been mapped across lifespan in rats and has a range of approximately 5 × 10^4^ to 10 × 10^4^ OSNs/mm^2^ (37). Comparatively, the mature OSN density measured in a limited set of human tissue was approximately 3 × 10^4^ OSNs/mm^2^ (49). While the human OSN density appears to be lower than that of rats, [^11^C]GV1-57 measures rat OSN population at younger ages when the OSN density is likely similar to that of humans. This comparison supported the translation of GV1-57 to NHPs, wherein we completed pilot autoradiography and imaging studies.

**Figure 6:**
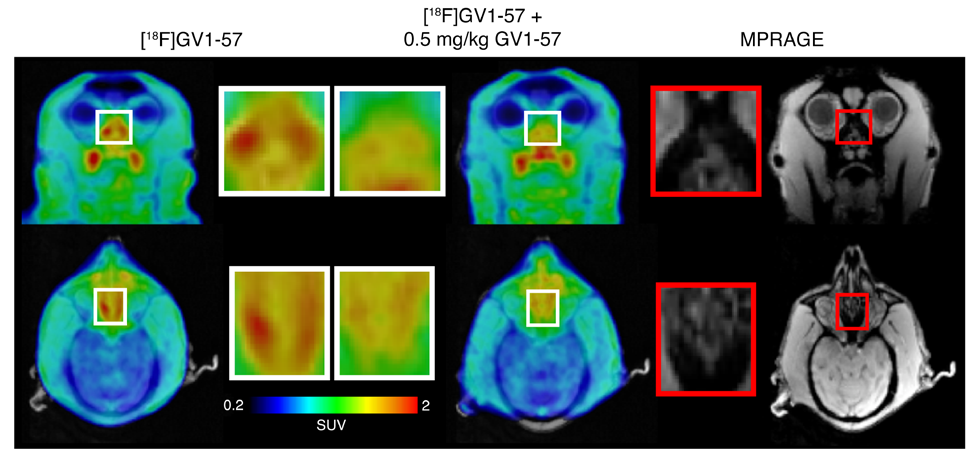
[^18^F]GV1-57 exhibits saturable nasal epithelium uptake in a rhesus macaque. Left: Coronal and axial [^18^F]GV1-57 PET images (SUV, NIH+white, 16–40 minutes) at the location of the superior nasal epithelium following administration of [^18^F]GV1-57 (4.89–5.07 mCi) with or without i.v. cotreatment with nonradiolabeled GV1-57 (0.5 mg/kg). Right: MPRAGE MR coronal and axial images at the location of the superior nasal epithelium. The red boxes shown on the MRI are equivalent regions to the areas boxed in white on the PET images.

To assess the uptake of GV1-57 within primate nasal epithelium, autoradiography was completed with tissue dissected from the nasal septum of a male baboon (*Papio anubis*). Pretreatment with nonradiolabeled GV1-57 (10 μM) reduced the intensity of the [^11^C]GV1-57 autoradiogram in the septal tissue, suggesting saturable binding of GV1-57 in NHP nasal tissue (Supplemental Figure 14). In vivo assessment of GV1-57 binding in NHPs was completed through administration of [^18^F]GV1-57 to a rhesus macaque in the presence or absence of nonradiolabeled GV1-57 (0.5 mg/kg). Within the superior nasal cavity, the location of OSNs within the primate nasal cavity (50), the [^18^F]GV1-57 PET images indicate a reduction in [^18^F]GV1-57 uptake following administration of nonradiolabeled GV1-57 (Figure 6). This suggests maintenance of saturable binding for GV1-57 within the NHP nasal epithelium in vivo. In concert with the PET imaging, a structural magnetic resonance image of the nasal cavity and brain was obtained using a T1-weighted magnetization-prepared rapid acquisition with gradient echo (MPRAGE) sequence. The MPRAGE provided structural information for comparison with the PET images, confirming that the superior nasal cavity is the region of saturable [^18^F]GV1-57 uptake (Figure 6). Together, these ex vivo and in vivo data highlight the translational potential for this new imaging tool, opening the door for exploration of OSN population flux in human development, aging, and disease.

## Discussion

GV1-57 is a first-in-class mature OSN population radiotracer for accurate in vivo OSN population measurements in mice and rats. Immunohistochemical and immunoblot validation of GV1-57 imaging outcomes affirms GV1-57 as a novel imaging biomarker for alterations in the level of mature OSNs resulting from growth, neuron recovery, aging, acute injury, or disease. We propose that the sensitivity of mature OSN levels, particularly to early stages of aging and disease, is partly a result of the high adult turnover of OSNs. The OE has rates of cellular proliferation of approximately 1 million new cells per day at P21 and about 70,000 new cells per day at P333 in rats (39), and the ensuing OSN turnover is several hundred- to several thousand-fold higher than the levels in the dentate gyrus or subventricular zone across lifespan (51, 52). This high neuron turnover yields a mature OSN population that is continuously impacted by fluctuations in neuron life cycle stages, including proliferation, differentiation, maturation, and death. Alterations in neuron health that impact the neuron life cycle stages will quickly emerge as an alteration of OSN population. Thus, we propose a model wherein OSN population correlates with homeostatic regulation of cerebral neuron population, and that the unique OSN properties potentially afford improved sensitivity to alterations of neuron population relative to the brain.

From a clinical perspective, the animal models used throughout our studies simulate disease states that may benefit in areas of therapeutic development, diagnosis, and treatment monitoring from an objective OSN population measurement. Traumatic brain injury results in hyposmia or anosmia in 26% to 69% of patients (53, 54) due to olfactory nerve shearing, which is simulated by anterior olfactory bulbectomy. Meanwhile, upper respiratory viral infections, which can lead to smell loss, are simulated by the ZnSO_4_ chemical ablation model. We hypothesize that use of bulbectomy and ZnSO_4_ models in combination with GV1-57 imaging and novel OSN regenerative therapeutics would assist development of first-in-class therapeutics for acute, trauma-induced smell loss.

Switching from acute injury to chronic models, our aged rodent imaging simulates human aging, and the tauopathy model simulates various neurodegenerative diseases. The OSN population deficits detected during aging and in the tauopathy model were detected during early stages of aging and disease, indicating early-stage sensitivity of the OSN population to global neuron dysregulation. In the aging model, the detected OSN efflux suggests OSN neurodegeneration may correlate with the aging-associated olfactory dysfunction that predicts increased mortality rates (17, 18). Monitoring this process in humans may provide insight into individual health and help guide medical interventions targeted at increasing healthy lifespan. The detection of early-stage OSN deficits in the tauopathy model suggests a potential use for GV1-57 OSN population monitoring in neurodegenerative disease, wherein GV1-57 may serve as an adjunct diagnostic or method for monitoring therapeutic response through measurement of OSN regeneration. Although initial NHP studies support the human translational potential of GV1-57, human studies, including characterization of and possible correction for partial volume effects, are needed to support application of GV1-57 to human OSN measurement. Since many of the use-models for GV1-57 benefit from longitudinal imaging of the OSN population, or monitoring OSN population flux, we propose the name Neuroflux for this radiotracer.

## Methods

### General synthetic methods.

Chemical reagents were American Chemical Society–grade purity or higher, and were used as received without further purification. Reactions were performed under inert atmosphere of nitrogen with standard Schlenk technique in oven-dried glassware. Analytical TLC was performed on TLC silica Gel 60-F254 plates (SiliCycle Inc.) with visualization by UV irradiation at 254 nm. Manual flash column chromatography was performed with SiliaFlash P60 silica gel (SiliCycle Inc.) and analytical-grade solvents, used without further distillation. NMR spectra were recorded at 22°C on a Varian 500-MHz spectrometer (^1^H, 500.16 MHz, and ^13^C, 125.784 MHz). ^1^H and ^13^C NMR chemical shifts are reported as δ in units of parts per million using residual solvent signals for referencing. Liquid chromatography-mass spectrometry (LCMS) analysis of organic synthetic reactions was conducted on an Agilent 1100 series high-performance liquid chromatograph with an attached Agilent 6310 ion trap mass spectrometer with an electrospray ionization source.

### Compound 2.

Compound 2 was synthesized according to conditions modified from the literature (55). Briefly, 6-chloro-2-fluoro-9*H*-purine (750 mg, 4.35 mmol, 1 Eq) was dissolved in *n*-butanol (50 ml) before addition of diisopropylethylamine (DIEA; 0.575 ml, 5.22 mmol, 1.21 Eq). The resulting solution was cooled to 0°C, and benzylamine (2 ml, 11.5 mmol, 2.64 Eq) was added dropwise over 25 minutes. The reaction mixture was stirred in the ice-water bath until it melted, at room temperature (RT) overnight, and then at 60°C for 6 hours. The reaction was allowed to cool to RT before being added to a mixture of ethyl acetate (EtOAc, 150 ml) and deionized (DI) water (100 ml). After shaking, the water layer was collected and the organic phase was washed once with DI water (100 ml), followed by brine (75 ml). The organic phase was dried over sodium sulfate and concentrated to give the pure product, *N*-benzyl-2-fluoro-9*H*-purin-6-amine (970 mg, 92%), as a pale yellow solid. LCMS (electrospray ionization): calculated for [C_12_H_10_FN_5_+H]^+^: 244, found 244 [M+H]^+^. ^1^H NMR (500 MHz, DMSO-d6): 4.64 (m, 2H), 7.23 (t, 5H, J = 7.1), 7.37–7.29 (m, 4H), 8.11 (s, 1H), 8.72 (s, 1H, NH), 12.93 (s, 1H, NH). ^13^C NMR (126 MHz, DMSO-d6): 43.2, 126.8, 127.3, 128.3, 139.3, 139.5, 155.3, 157.8, 159.5.

### Compound 3, nonradiolabeled GV1-57.

Compound 2 (300 mg, 1.23 mmol, 1 Eq), potassium carbonate (852.4 mg, 6.17 mmol, 5 Eq), and methyl iodide (374 μl, 6.01 mmol, 4.89 Eq) were added to dimethylacetamide (DMA, 3.5 ml), and the reaction was stirred at RT for 7 hours. Excess methyl iodide was removed by first passing N_2_ (gas) through the solution for 15 minutes and then placing the reaction under vacuum. The reaction was then added to EtOAc (50 ml), which was washed with DI water (100 ml). The aqueous phase was collected and washed with EtOAc (3 × 50 ml). The combined organic phases were then washed with brine (50 ml), dried over sodium sulfate, and concentrated. After manual flash column chromatography (dichloromethane/EtOAc/methanol, 49:49:2), 215.5 mg (68%) of pure nonradiolabeled GV1-57 was obtained as a pale yellow solid. LCMS (electrospray ionization): calculated for [C_13_H_12_FN_5_+H]^+^: 258, found 258 [M+H]^+^. 1H NMR (500 MHz, DMSO-d6): 3.67 (s, 3H), 4.63 (d, 2H, J = 6), 7.20–7.33 (m, 5H), 8.10 (s, 1H), 8.84 (s, 1H, NH). 13C NMR (100 MHz, DMSO-d6): 29.5, 43.2, 117.3, 126.8, 127.2, 128.2, 139.3, 141.8, 150.4, 150.6, 155.7, 155.9, 157.7, 159.7.

### Compound 3, [^11^C]GV1-57.

DMSO (300 μl) was added to a vial containing compound 2 (1–1.5 mg) and potassium hydroxide (4–6 mg, powdered). The solution was transferred to a reaction vessel with a stir bar, and the reaction vessel was placed in a TRACERlabFX-M synthesizer reactor. The automated system was used to add [^11^C]methyl iodide (100–400 mCi), and the solution was stirred at 80°C for 5 minutes. Quenching solution was then added (1.7 ml 35% acetonitrile [ACN] and 65% H_2_O with 0.065% formic acid), and the solution (2 ml) was injected onto a Phenomenex Luna C18 semipreparative column. The product was purified via HPLC at a flow rate of 5 ml/min using isocratic conditions (35% ACN and 65% H_2_O with 0.065% formic acid). The desired radioactive fraction was collected, and the product was reformulated in 10% ethanol in saline (10 ml). The identity of the product was confirmed using liquid chromatography under standard gradient conditions with a coinjection of the radioactive product and a standard solution containing nonradiolabeled GV1-57.

### Animals.

Male Sprague-Dawley rats (Charles River Laboratories) were pair-housed until they reached a weight of 500 g, and female mice (Jackson Laboratory or Taconic) were singly or group-housed, as received. All animals were kept on a 12 hours/12 hours light-dark cycle, switching at 7 am and pm. All treatments and imaging were performed according to procedures approved by the IACUC.

### General rodent imaging procedure.

Before imaging, animals were anesthetized with inhalational isoflurane, and a catheter was placed in a lateral tail vein. An extension line was used to attach the catheter to a syringe with heparinized saline. Animals were then placed in 1 of 2 instruments (a GammaMedica Triumph PET/CT/SPECT scanner or a Siemens R4 PET scanner) and maintained under inhalational isoflurane anesthesia for [^11^C]GV1-57 injection, a 60-minute dynamic PET scan, and a 5-minute attenuation or CT scan. Following imaging, animals were placed in their cages and monitored until they recovered from the anesthesia.

### General rodent image processing procedure.

All PET data were histogrammed using the following time slices: 8 × 15 seconds, 8 × 60 seconds, 10 × 120 seconds, and 6 × 300 seconds. For scans completed in the Triumph scanner, CT images of the animals were taken for PET image attenuation. For animals imaged in the R4, a Cobalt (Co) 57 line source was used to create an attenuation correction map. Reconstructed PET data were analyzed in Amide. Rat PET data were first aligned with CT data from an age-matched rat with all animals from a single experiment and/or age-group overlaid. A single VOI was then placed over the OE, centered on the region of highest uptake. A second VOI was placed over the brain for use as the reference region for Logan analysis. Mouse PET data were aligned with the corresponding individual mouse CT, or an experimentally matched CT. VOIs with consistent dimensions were then placed over the OE and brain of individual mice for Logan analysis. DVRs and binding potentials were calculated using a t* of 45 minutes. Voxel-wise SUV (mean radioactivity/injected dose/weight) maps were calculated, and images were generated for 3–45 minutes after [^11^C]GV1-57 administration.

### [^11^C]GV1-57 saturable binding and in vivo IC_50_.

To analyze saturable binding, 2-month-old Sprague-Dawley rats were injected with nonradiolabeled GV1-57 (16 mg/kg at 4 mg/ml in 1:1:8 DMSO/Tween-20/saline, *n* = 3) or vehicle (1:1:8 DMSO/Tween-20/saline, *n* = 3) 5 minutes before injection of [^11^C]GV1-57 (0.93 ± 0.08 mCi, in 1:9 ethanol/saline). To determine the in vivo IC_50_, nonradiolabeled GV1-57 (0, 0.25, 0.5, 1, 2, and 4 mg/kg, *n* = 1 per dose) and [^11^C]GV1-57 (0.51 ± 0.02 mCi) were coadministered to animals in 1 ml of 1:1:1:7 DMSO/Tween-20/ethanol/saline.

### [^11^C]GV1-57 imaging following anterior bulbectomy.

Three-month-old female C57BL/6 mice were deeply anesthetized with isoflurane anesthesia for either a bilateral (*n* = 3) or a unilateral (*n* = 3) olfactory bulbectomy. The mice were placed in a stereotaxic head stage and given an s.c. injection of 0.1 ml of lidocaine (2%) before the incision was made. A midline incision was made between the orbits to expose the region of the skull above the olfactory bulbs. One or two cranial windows, each approximately 1 mm in diameter, were cut with a drill over the anterior portion of the olfactory bulbs. The anterior portion of the olfactory bulb (~10% of the total bulb) was removed by slicing the neural tissue with a needle blade and extracting it through the cranial window before suturing the wounds. Animals were allowed to recover on a heating pad for 15–30 minutes and given an injection of buprenorphine (0.05–0.1 mg/kg) for pain management. Once fully conscious, mice were housed individually and allowed to recover for 3 days and given buprenorphine injections every 12 hours. After recovery, mice were injected i.v. with [^11^C]GV1-57 (0.55 ± 0.053 mCi in 1:9 ethanol/saline) and imaged. Animals were imaged 3 days after bulbectomy, except for the bilateral animal with the highest DVR, which was imaged 2 days after bulbectomy. Following imaging, animals were euthanized, and their OE tissue was collected for immunoblotting (*n* = 2 per group).

### ZnSO_4_ treatment and longitudinal imaging.

Three-month-old male Sprague-Dawley rats were anesthetized with inhalational isoflurane for intranasal administration, while in a supine position, of either ZnSO_4_ (10% in saline, 100 μl per nostril, *n* = 7) or vehicle (saline, 100 μl per nostril, *n* = 4). ZnSO_4_ and vehicle were administered bilaterally, with injection in the right nostril occurring 15 minutes before injection of the left nostril. For the injection, anesthetized animals were placed in a supine position, with a slight tilt toward the side opposite the nostril being injected. PE 10 tubing attached to a syringe with the ZnSO_4_ or vehicle dose was then inserted 14 mm into the nostril, and the solution was quickly expunged into the nasal cavity. The head was then held at a 45° angle with the body supine for 1 to 2 seconds before the rat’s nose was held down to allow excess solution to drain from the nostril. Animals were allowed to wake between right and left nasal injections to assist with clearing of the nasal passages. Using this procedure, there was no animal mortality. From 1 to 74 days after ZnSO_4_ treatment, animals were imaged with [^11^C]GV1-57 (1.02 ± 0.14 mCi in 1:9 ethanol/saline). After the final imaging session, animals were euthanized, and their OE tissue was collected for immunoblotting.

### [^11^C]GV1-57 imaging of rat neurodevelopment.

At the specified ages, male Sprague-Dawley rats were injected i.v. with [^11^C]GV1-57 (0.93 ± 0.14 mCi, in 1:9 ethanol/saline) (1.3 months, *n* = 5; 2 months, *n* = 3; 3 months, *n* = 4; 5.5 months, *n* = 8; 9 months, *n* = 8; 12 months, *n* = 7; 15 months, *n* = 3). Some animals were additionally injected with vehicle (4 ml/kg; in 1:1:8 DMSO/Tween-20/saline) 5 minutes before radiotracer injection. Animals were imaged for 60 minutes.

### [^11^C]GV1-57 imaging during mouse neurodevelopment and aging.

At the specified ages, B6C3F1 or C57BL/6 female mice were injected i.v. with [^11^C]GV1-57 (0.54 ± 0.11 mCi, in 1:9 ethanol/saline) and imaged for 60 minutes (3 months, *n* = 6; 4.2 months, *n* = 2; 7 months, *n* = 3; 12 months, *n* = 5; 23 months, *n* = 3).

### [^11^C]GV1-57 imaging of rTg4510 mice.

Female rTg4510 and WT control mice were obtained from Taconic (provided by Eli Lilly and Co., Indianapolis, Indiana, USA). Groups of 4 animals of the specified ages and genotypes were injected i.v. with [^11^C]GV1-57 (0.51 ± 0.091 mCi, in 1:9 ethanol/saline) and imaged for 60 minutes.

### Rhesus macaque imaging and image processing procedure.

A paired baseline/blocking PET/MR study was performed on a male macaque (~11 kg) on 2 separate days. The study was approved by the IACUC at the Massachusetts General Hospital. The animal was deprived of food for 12 hours prior to the study. Anesthesia was induced with i.m. ketamine (10 mg/kg) and xylazine (0.5 mg/kg). For maintenance of anesthesia throughout the study, the animal was provided with 1%–1.2% isoflurane in oxygen while a dose of yobine (0.11 mg/kg, i.m.) was given to reverse the effects of ketamine/xylazine before the start of the scan. Vital signs including end-tidal CO_2_, O_2_ saturation, heart rate, and respiration rate were monitored continuously (recorded every 15 minutes) and were maintained within normal physiological ranges.

PET and MR images were acquired on a 3T Siemens TIM-Trio with a BrainPET insert (Siemens). A custom PET/MRI-compatible 8-channel array coil for NHP brain imaging was used to improve image signal and quality compared with using a clinical human head coil. Dynamic PET image acquisition was initiated, followed by i.v. administration of [^18^F]GV1-57 (4.89–5.07 mCi) as a manual bolus over approximately 30 seconds. A baseline [^18^F]GV1-57 PET scan was first carried out. A second PET scan (i.e., the blocking scan) was performed, on a separate day, with coinjection of [^18^F]GV1-57 and nonradiolabeled GV1-57 (0.5 mg/kg) to determine saturable binding. No physiological response to 0.5 mg/kg GV1-57 was observed. Dynamic PET data were collected and stored in list mode for 80–90 minutes. The PET images were reconstructed using the 3D ordinary Poisson expectation maximization algorithm (32 iterations) with detector efficiency, decay, dead time, attenuation, and scatter corrections applied. PET data were reconstructed with gradually increasing intervals (8 × 15 seconds, 8 × 60 seconds, 10 × 120 seconds, 10–12 × 5 minutes). The highest image resolution was on the order of 2–3 mm for BrainPET. The final image volumes were reconstructed into 76 slices with 128 × 128 pixels and a 2.5-mm isotropic voxel size. A high-resolution, T1-weighted, anatomical MRI scan using a multi-echo MPRAGE sequence (TR = 2,530 ms, TE1/TE2/TE3/TE4 = 1.64/3.5/5.36/7.22 ms, TI = 1,200 ms, flip angle = 7°, and 1 mm isotropic) was obtained before the injection of the radiotracer. The anatomical MRI images between the baseline and blocking scans were coregistered using a linear transformation. The transformation parameters were then applied to the simultaneously collected dynamic PET data. Voxel-wise SUV (mean radioactivity/injected dose/weight) maps were calculated for the baseline and blocking scan, and images were generated for 16–40 minutes after [^18^F]GV1-57 administration.

### OE dissection, rodent.

Animals were decapitated, and the skin and tissue around the skull were removed using a combination of dissection scissors and bone rongeurs. Forceps were used to clean the eye socket, and rongeurs used to cut both maxilla where they branch away from the nasal cavity. Rongeurs were then used to cut away the upper incisors and the very front of the nasal cavity. Rongeurs (rats) or scissors (mice) were used to cut away the occipital and interparietal bones, then used to cut along the lateral edges of the parietal and then frontal bones to reach the nasal cavity. Superior nasal bones were removed, then the side of the nasal cavity to expose the OE. Non-OE tissue was removed with forceps. For IHC, scissors were used to cut the intact, whole OE away from the palate. For immunoblotting of rat OE, small forceps were used to pull out the turbinate bones and associated OE. The OE located on the septum was then carefully removed and placed in an Eppendorf tube and snap-frozen on dry ice. For immunoblotting of mouse OE, whole OE was collected and snap-frozen over dry ice. For IHC, mice were perfused with 4% paraformaldehyde (PFA; P6148, Sigma-Aldrich) in PBS.

### Tissue lysate preparation.

Rodent OE tissue, septum only (rat) or whole OE (mouse), was lysed at 100 mg/ml in lysis buffer containing PBS, 0.15% NP-40, and a protease inhibitor cocktail (04693159001; Roche). The tissue was homogenized for 1 minute with an electric mortar, and the homogenized tissue, on ice, was sonicated (model CL-18, Fisher Scientific) at 50% power for 30 one-second pulses. Lysate was incubated with rotation at 4°C for 15 minutes and centrifuged at 18,000 *g* at 4°C for 20 minutes. The supernatant was collected, and total protein concentration was determined using a BCA protein assay (23227, Pierce).

### Immunoblotting.

Samples were each diluted to a concentration of 1.5 μg/μl, mixed with one-third volume of 3× SDS loading buffer (B7703S; NEB), and heated to 95°C for 10 minutes. Ten micrograms of total protein was loaded in each lane and separated on Criterion Stain-Free 4%–20% gels (567-8095; Bio-Rad) at 150 V for 70 minutes. Proteins were transferred to Low Fluorescence PVDF membrane (162-0264; Bio-Rad) at 0.36 A for 60 minutes. Gels and membranes were imaged with a Chemidoc XRS system (170-8265; Bio-Rad) to ensure equal lane loading and transfer. Membranes were blocked in TBST (pH 7.4, 0.2% Tween-20) containing 5% skim milk (170-6404; Bio-Rad) at 4°C overnight followed by washing in TBST at RT (rest of protocol performed at RT). Primary and secondary antibody incubation was as follows: OMP (1:80,000; 544-10001; Wako) followed by donkey anti-goat–HRP (1:10,000 in 3.5% BSA in TBST; sc2020; Santa Cruz Biotechnology Inc.); GAP43 (1:2,000; NBP1-41123; Novus Biologicals) followed by donkey anti-sheep–HRP (1:10,000 in 3.5% BSA in TBST; ab97125; Abcam); REEP6 (1:1,000; 12088-1-AP, Proteintech) followed by donkey anti-rabbit–HRP (1:5,000; 7074S; Cell Signaling Technology). Membranes were incubated and washed in TBST, followed by incubation with ECL prime detection substrate (RPN2232; GE Healthcare), and imaging with a Chemidoc XRS system. Band intensity and background measurements were obtained in Image Lab 5.1 (Bio-Rad) using the volume tool. Relative band intensity per microgram of protein was normalized to control group or the youngest age group. To calculate total cellular marker protein in each tissue sample, the relative band intensity was multiplied by the total protein extracted from the tissue sample.

### Autoradiography: rat.

Freshly dissected, whole rat OE was removed and submerged in OCT mounting medium (25608-930; VWR) followed by snap freezing on dry ice. Frozen, embedded tissue was sliced in 10-μm coronal sections onto slides (4 sections per slide) using a cryostat (HM550; Thermo Fisher Scientific). Slides were immersed in 4% PFA in phosphate buffer (BM-698, Boston Bioproducts) containing 2% EtOH for 30 minutes followed by 10 mM Tris, pH 7.5, at 4°C for 10 minutes. Sections were then submerged for 20 minutes in 10 mM Tris buffer containing nonradiolabeled GV1-57 (10, 5, 0.5, 0.1, 0 μM) and 5% DMSO. To these solutions was added [^11^C]GV1-57 (~200 nCi), before incubation at RT for 12 minutes. Slides were then washed in 10 mM Tris buffer for 10 minutes at RT and dried under vacuum for 30 minutes at 25°C. All slides were exposed to multisensitive phosphor screens (PerkinElmer) for 1 hour and imaged with a Cyclone Plus Storage Phosphor system (PerkinElmer). Images were colored using the Rainbow lookup table in ImageJ (NIH) with equivalent thresholds for brightness. Intensity values for each OE slice were measured using ImageJ, background subtracted, and averaged across replicate sections. All values were normalized to those of the DMSO-treated OE sections and displayed using GraphPad Prism software.

### Autoradiography: Papio anubis.

A freshly dissected *Papio anubis* nasal cavity was placed in freshly made 4% PFA in PBS and stored at 4°C. Tissue from either side of the nasal septum was carefully detached from the septum using forceps and placed in 10 mM Tris, pH 7.5, at 4°C. The tissue was then submerged for 20 minutes in 10 mM Tris buffer containing nonradiolabeled GV1-57 (10 or 0 μM) and 5% DMSO. To these solutions was added [^11^C]GV1-57 (~200 nCi), before incubation at RT for 15 minutes. The septal tissue was washed quickly by submerging 5 times in 10 mM Tris buffer and then transferred to a conical vial containing fresh 10 mM Tris buffer for 10 minutes at RT. The tissue was removed, air-dried, and exposed to a multisensitive phosphor screen (PerkinElmer) for 1 hour and imaged with a Cyclone Plus Storage Phosphor system (PerkinElmer). Images were colored using the color scale option in OptiQuant software (PerkinElmer).

### OE thickness measurements.

Whole OE collected from mice for IHC purposes was fixed in 4% PFA (P6148; Sigma-Aldrich) in PBS for 24 hours, cryoprotected in 30% sucrose in PBS for 48–72 hours, snap-frozen in M-1 embedding matrix (1310; Thermo Fisher Scientific), and stored at –80°C. Embedded OE was sliced coronally (20 μm; HM550; Thermo Fisher Scientific), with sections thaw-mounted onto microscope slides (12-550-18; Thermo Fisher Scientific). Before DAPI staining, slides were thawed, dried at RT for 15 minutes, postfixed in 4% PFA in PBS for 10 minutes, washed in PBS (2 × 10 minutes), permeabilized in PTS (PBS plus 0.5% Triton X-100) for 10 minutes at RT, and washed in PBS (3 × 10 minutes). Excess PBS was removed from slides using a Kimwipe (Kimtech Science) before addition of DAPI-containing mounting medium (150 μl per slide; P-36931, Life Technologies) and placement of coverglass. Slides were air-dried overnight at RT and sealed with clear nail polish. Using an inverted epifluorescence microscope (Axio Observer.Z1; Zeiss) and camera (Exi-Aqua; Q-Imaging), images of the OE were taken with a ×10 Plan-Apochromat lens, centered on the top or middle portion of the septal OE. Images were acquired using MetaMorph software, version 7.7.4.0. Sections from WT and rTg4510 animals were paired by OE depth and region within the septum (top or middle). OE thickness measurements were made in ImageJ, wherein paired regions (WT and rTg4510, within age) were bounded by lines and measurements were made approximately every 10 μm within the boundaries. All measurements within the boundaries were averaged to provide final thickness measurement. Thickness measurements were then compared based on pairings of OE depth and septal region.

### Tau pathology.

Formalin-fixed paraffin-embedded brains from female rTg4510 mice at 2, 4, 6, and 8 months of age were sectioned (6 μm thickness) in the sagittal plane. IHC was performed using a phosphospecific tau antibody (PG-5, pSer409; gift from Peter Davies, Feinstein Institute for Medical Research, Manhasset, New York, USA) as previously described (56). Stained sections were digitized using the Scanscope AT slide scanner (Aperio) at ×20 magnification. Imagescope software (v11.1.2.780; Aperio) was used to view the digitized tissue sections.

### Assessment of [^11^C]GV1-57 images from individual WT and rTg4510 mice.

A contingency table was developed for analysis of the WT and rTg4510 animals using animal genotype as the reference standard. Sixty-minute scans from individual animals were converted into SUV and averaged from 3–45 minutes. A training slide was developed for each age group (3.7 and 7 months), showing an example WT and rTg4510 image, including details of the SUV thresholds (0–2.15 for 3.7 and 7 months). Reviewers were asked to view individual animal scans in Amide, with the ability to assess scans in 3D space and adjust the SUV thresholds. Two radiologists were asked to come to a consensus agreement of each animal’s genotype. Given the small sample size (6 animals per age), the percentage of correct classifications was calculated instead of a χ^2^ statistical analysis being completed. Scans used for this analysis were taken from a single instrument, the Triumph scanner, which represented a majority of the scans acquired. The trained radiologists who read the scans have a combined 37 years of experience with small-animal imaging and/or clinical image assessment.

### IC_50_ and correlation analyses.

IC_50_ values were determined using GraphPad Prism software with the log(inhibitor) versus response (3 parameters) nonlinear fit option. Spearman correlations were performed using GraphPad Prism.

### Statistics.

All data are presented as the mean ± SEM and were compared using a paired or unpaired, 1- or 2-tailed Student’s *t* test with a Bonferroni correction to account for multiple comparisons when necessary. For Figure 2B, a 1-way ANOVA was followed by a post hoc 1-tailed Student’s *t* test between control and bilateral bulbectomy groups. For Figure 3C, the software package R was used to complete a regression analysis, which was assessed by a type III ANOVA with Satterthwaite approximation. For single intergroup comparisons, a *P* value less than 0.05 was considered significant; for 2 intergroup comparisons, a *P* value less than 0.025 was considered significant (Bonferroni correction).

### Study approval.

All rodent and nonhuman primate studies were approved by the IACUC at the Massachusetts General Hospital.

## Author contributions

GCV, JMH, and MWA designed the project. GCV, MMR, LC, MWA, and JMH designed experiments. GCV, MMR, LC, JE, SPH, ELR, HYW, MJO, ZA, TKM, JES, CW, and FAS performed experiments. GCV designed and synthesized the radiotracer and analyzed imaging data with input from HYW and JMH. GCV, MMR, JE, and SPH analyzed immunoblotting, autoradiography, and microscopy data. GCV, MMR, MWA, and JMH interpreted the data. GCV, MMR, JE, SPH, HYW, and ZA wrote the Methods and supplemental information. MJO, ZA, and TKM supplied the rTg4510 animals, and designed and led the cross-sectional tau pathology characterization of the rTg4510 brain sections. GCV, ELR, MWA, and JMH wrote the manuscript with input from all authors.

## Supplementary Material

Supplemental data
