# Supplementary material for: Nasal neuron PET imaging quantifies neuron generation and degeneration
Source: J Clin Invest. 2017 Jan 23;127(2):681–94. doi: 10.1172/JCI89162 (PMC5272198; doi:10.1172/JCI89162)
Supplement: Supplemental data [file jci-127-89162-s001.pdf]

Supplemental Information for

Nasal neuron PET imaging quantifies neuron generation and degeneration

Genevieve C. Van de Bittner, Misha M. Riley, Luxiang Cao, Janina Ehses, Scott P. Herrick, Emily L. Ricq, Hsiao-Ying Wey, Michael J. O'Neill, Zeshan Ahmed, Tracey K. Murray, Jaclyn E. Smith, Changning Wang, Frederick A. Schroeder, Mark W. Albers, Jacob M. Hooker

Tables and Figures

Supplemental Table 1 (appended to the end of the SI). GV1-57 does not show broad-spectrum kinase inhibition. A comprehensive kinase screen was completed by Reaction Biology Corp. using 5 μM non-radiolabeled GV1-57 in the presence of 1 μM ATP. Duplicate measurements were acquired against each kinase. The control compound, Staurosporine, was tested in the 10-dose IC_{50} mode with 4-fold serial dilutions starting at 20 or 100 μM. Alternate control compounds were tested in the 10-dose IC_{50} mode with 3-fold serial dilutions starting at 20 or 100 μM. For most kinases, there was no inhibition of kinase activity. The highest inhibition was of homeodomain interacting protein kinase 4 (HIPK4), with 51% inhibition at a concentration 15-fold higher than the measured ex vivo [^{11}C]GV1-57 IC_{50} (Supplemental Figure 5).

Supplemental Table 2. GV1-57 does not inhibit binding interactions with adenosine and purinergic receptors. Adenosine and purinergic receptor inhibition assays were completed by Eurofins Panlabs using 0.5 and 1.0 μM non-radiolabeled GV1-57. Eurofins Panlabs assays were binding displacement assays, and no significant inhibition of binding was detected. Labeled molecules used for determination of binding displacement were as follows: 1 nM [^{3}H]DPCPX (A1), 50 nM [^{3}H]CGS-21680 (A2A), 1.6 nM [^{3}H]MRS1754 (A2B), 0.5 nM [^{125}I]AB-MECA (A3), 8 nM [^{3}H]α, β-Methylene-ATP (P2X, general), 0.1 nM [^{35}S]ATP-αS (P2Y, general).

Receptor	Percent Inhibition*
A1	3 (0.5), -9 (1.0)
A2A	1 (0.5), 3 (1.0)
A2B	4 (0.5), 1 (1.0)
A3	-7 (0.5), -6 (1.0)
P2X	6 (0.5), -7 (1.0)
P2Y	-9 (0.5), -1 (1.0)

*Percent inhibition: ± percent inhibition (non-radiolabeled GV1-57, μM)

Supplemental Table 3. Individual olfactory sensory neuron influx rates during normative development. Rates (DVR/month) were calculated for individual rats. Between 5.5 and 12 months of age, rates of OSN population influx are significantly higher (P < 0.005) for animals starting with a DVR less than 5.5 compared to animals starting with a DVR greater than 6.0. The starting DVR is the DVR at 5.5 months of age.

Animal	Rate[a] (DVR/month)	DVR[b] (at 5.5 months)
1	0.084	6.76
2	0.30	5.38
3	0.23	5.04
4	0.26	4.40
5	0.12	6.24
6	0.11	6.92

[a] Calculated from 5.5–12 months of age
[b] t^* = 45 min

Supplemental Table 4. Olfactory sensory neuron regrowth rates following zinc sulfate tissue ablation. OSN regrowth rates (DVR/month) after $ZnSO_4$ treatment were calculated using the lowest post-$ZnSO_4$ DVR (lowest OSN population), the highest post-$ZnSO_4$ DVR (highest OSN population), and the intervening time interval between these DVR measurements. The OSN regrowth rates vary considerably across individual animals, with a 3.7-fold difference between the lowest and highest rate.

Animal	Rate (DVR/month)
1	0.55
2	1.5
3	0.96
4	2.0

Supplemental Table 5. Contingency table for classification of WT and rTg4510 animals at 3.7 months of age. Two trained radiologists used [^{11}C]GV1-57 images (SUV, 0–2.15) to classify individual 3.7 month old animals as having high or low radiotracer uptake in the olfactory epithelium (OE). The reference standard (*i.e.* gold standard) for this experiment was animal genotype (*n* = 3 per group).

Genotype / [^{11}C]GV1-57 Image, 3.7 mo	Positive genotype, rTg4510	Negative genotype, WT
Positive scan, low uptake	3	1
Negative scan, high uptake	0	2

Supplemental Table 6. Contingency table for classification of WT and rTg4510 animals at 7 months of age. Two trained radiologists used [^{11}C]GV1-57 images (SUV, 0–2.15) to classify individual 7 month old animals as having high or low radiotracer uptake in the OE. The reference standard (*i.e.* gold standard) for this experiment was animal genotype (*n* = 3 per group).

Genotype / [^{11}C]GV1-57 Image, 7 mo	Positive genotype, rTg4510	Negative genotype, WT
Positive scan, low uptake	3	0
Negative scan, high uptake	0	3

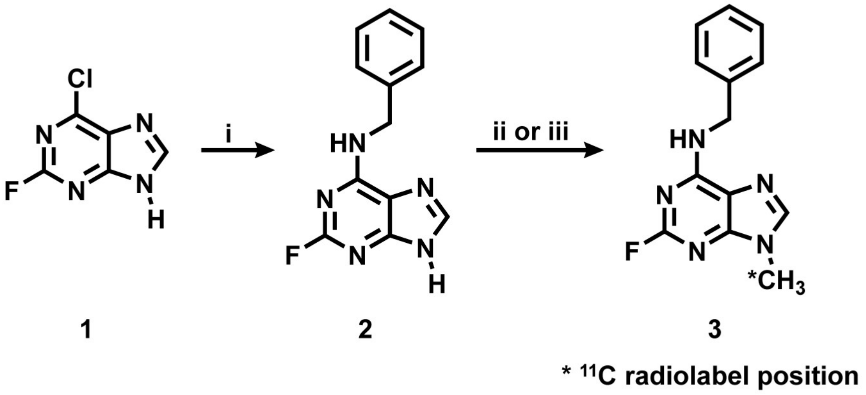

Supplemental Figure 1. Synthesis of non-radiolabeled GV1-57 and [^{11}C]GV1-57. Conditions: (i) Benzylamine, DIEA, *n*-BuOH, RT – 60 °C, 30 h, 92%. (ii) MeI, K_2CO_3, DMA, 7 h, 68%. (iii) [^{11}C]MeI, KOH, DMSO, 80 °C, 5 min, 14.2 ± 1.5% RCY, not decay corrected.

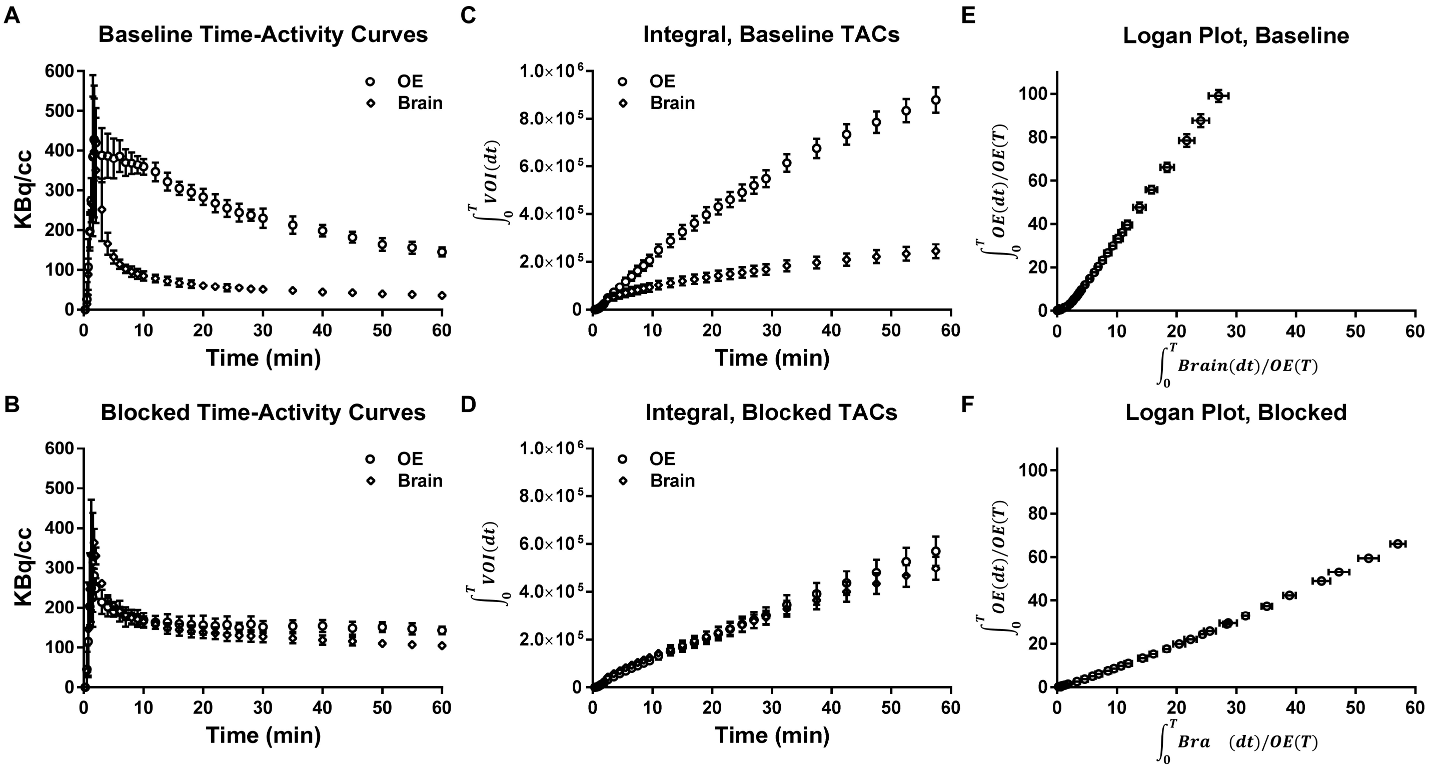

Supplemental Figure 2. Graphical representation of [^{11}C]GV1-57 Logan analysis. (**A, B**) Averaged time-activity curves (TACs) for the OE and the brain after injection with [^{11}C]GV1-57 (Baseline, **A**) or [^{11}C]GV1-57 and 16 mg/kg non-radiolabeled GV1-57 (Blocked, **B**). (**C, D**) Averaged integrals of the OE and brain TACs for animals injected with [^{11}C]GV1-57 (Baseline, **C**) or [^{11}C]GV1-57 and 16 mg/kg non-radiolabeled GV1-57 (Blocked, **D**). VOI y-axis label refers to either the OE or brain volume of interest. (**E, F**) Logan plots for animals injected with [^{11}C]GV1-57 (Baseline, **E**) or [^{11}C]GV1-57 and 16 mg/kg non-radiolabeled GV1-57 (Blocked, **F**). Treatment with 16 mg/kg non-radiolabeled GV1-57 occurred 5 min prior to [^{11}C]GV1-57 injection. Error bars are ± SEM, n = 3 for baseline and blocked groups.

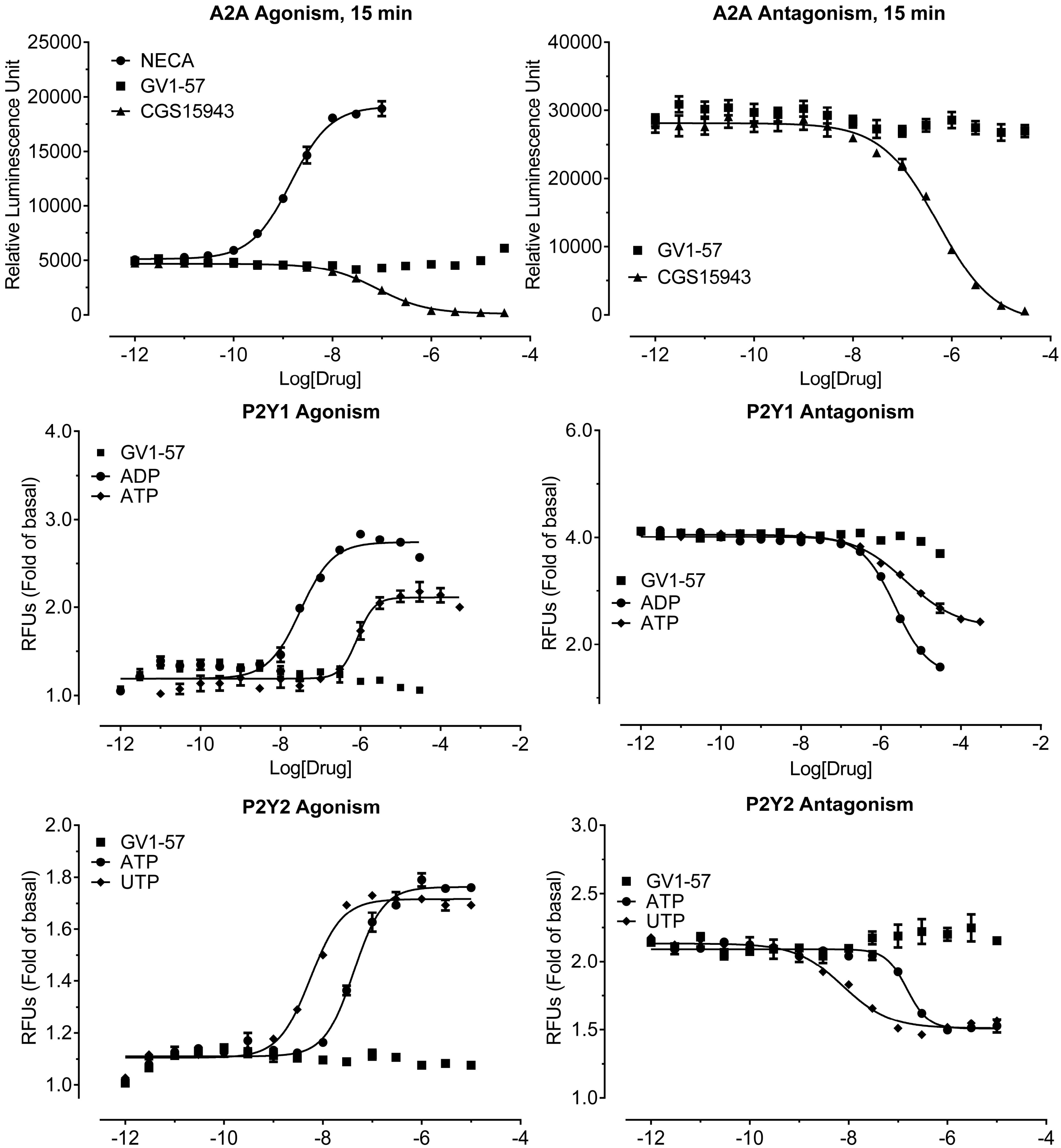

Supplemental Figure 3. GV1-57 does not inhibit activity of select adenosine and purinergic receptors. Adenosine (A2A) and purinergic (P2Y 1 and 2) receptor functional agonism and antagonism assays were completed by the PDSP using 3 pM – 30 μM non-radiolabeled GV1-57 (3, 10, 30, 100, 300 pM; 1, 3, 10, 30, 100, 300 nM; 1, 3, 10, 30 μM) (30). The data indicates no GV1-57 agonism or antagonism at these receptors. To desensitize the receptors, agonists were used in the antagonist functional assays, as follows: 30 nM NECA for A2A, 3 μM ADP for P2Y1, and 100 nM ATP for P2Y2.

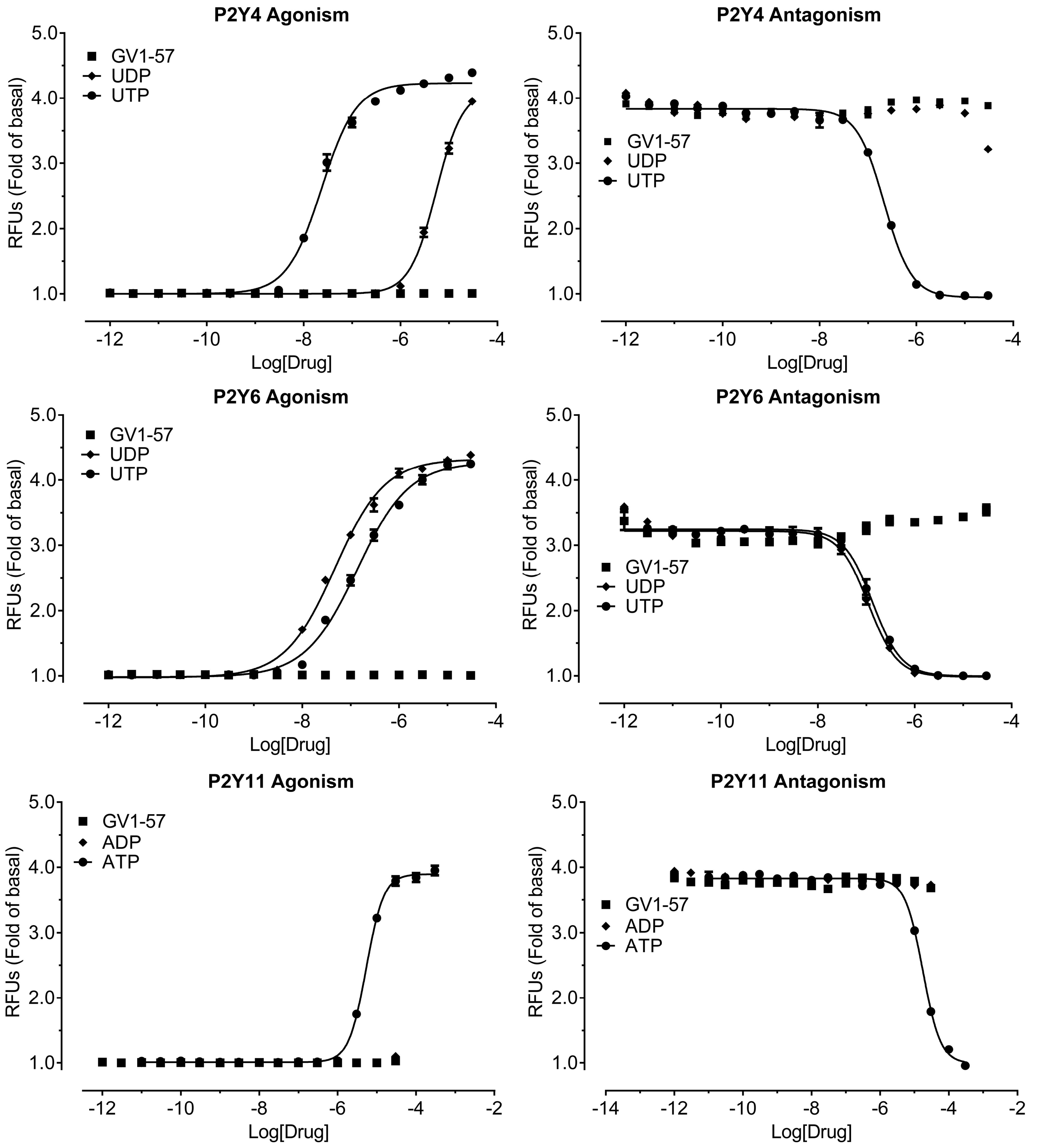

Supplemental Figure 4. GV1-57 does not inhibit activity of select adenosine and purinergic receptors. Purinergic (P2Y 4, 6, and 11) receptor functional agonism and antagonism assays were completed by the PDSP using 3 pM – 30 μM non-radiolabeled GV1-57 (3, 10, 30, 100, 300 pM; 1, 3, 10, 30, 100, 300 nM; 1, 3, 10, 30 μM) (30). The data indicates no GV1-57 agonism or antagonism at these receptors. To desensitize the receptors, agonists were used in the antagonist functional assays, as follows: 300 nM UTP for P2Y4, 100 nM UDP for P2Y6, and 100 μM ATP for P2Y11.

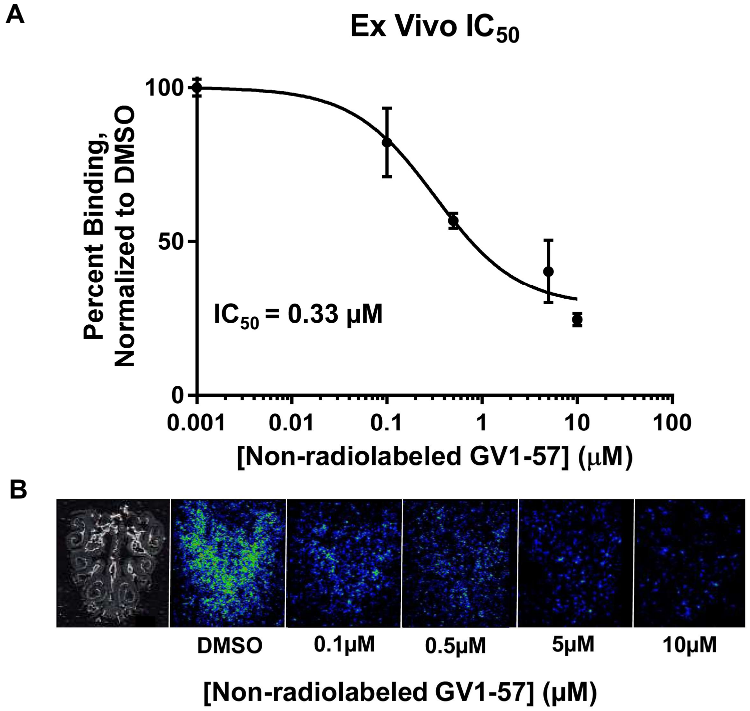

Supplemental Figure 5. [^{11}C]GV1-57 binding to olfactory epithelium sections is blocked by non-radiolabeled GV1-57. (**A**) Quantification of [^{11}C]GV1-57 autoradiography with coronal rat OE sections (10 μm) following a 20 min pre-incubation with non-radiolabeled GV1-57 (0–10 μM). Error bars are ± SEM; *n* = 8 sections/concentration. (**B**) Representative images of [^{11}C]GV1-57 binding to rat OE sections following pre-incubation with specified non-radiolabeled GV1-57 concentrations. Image on left is a digital scan of a representative coronal rat OE section that has been placed on a microscope slide.

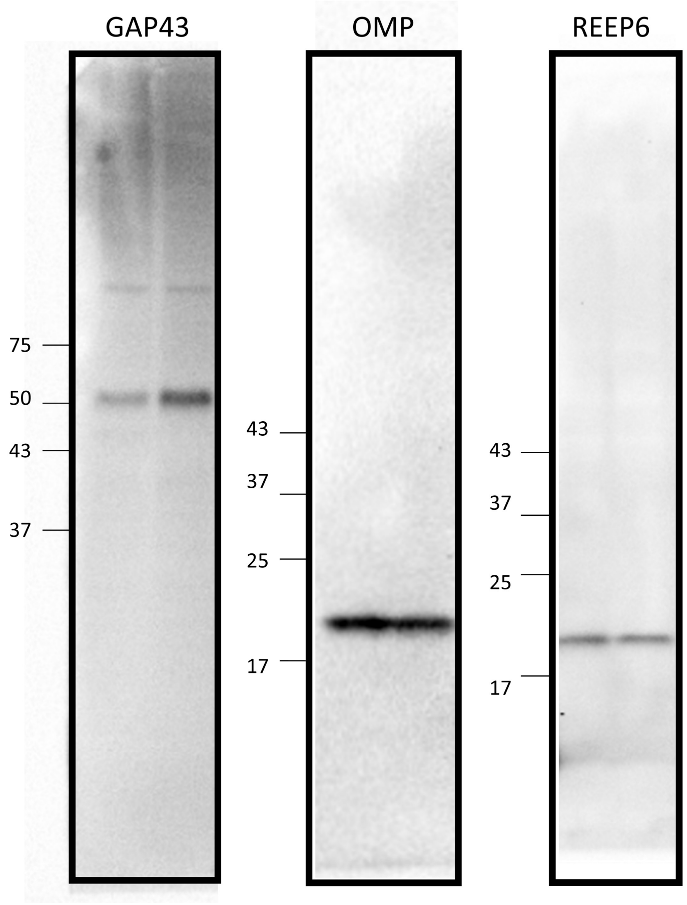

Supplemental Figure 6. Representative immunoblots for the cell-type-specific olfactory epithelium markers. Full immunoblots of rodent OE lysate indicate good selectivity of the chosen antibodies for anticipated protein molecular weights for GAP43 (25 kDa, apparent molecular weight ~43 kDa) (35), OMP (19 kDa), and REEP6 (22 kDa). The immunoblots shown in this figure are also shown in the left panel of Figure 4C.

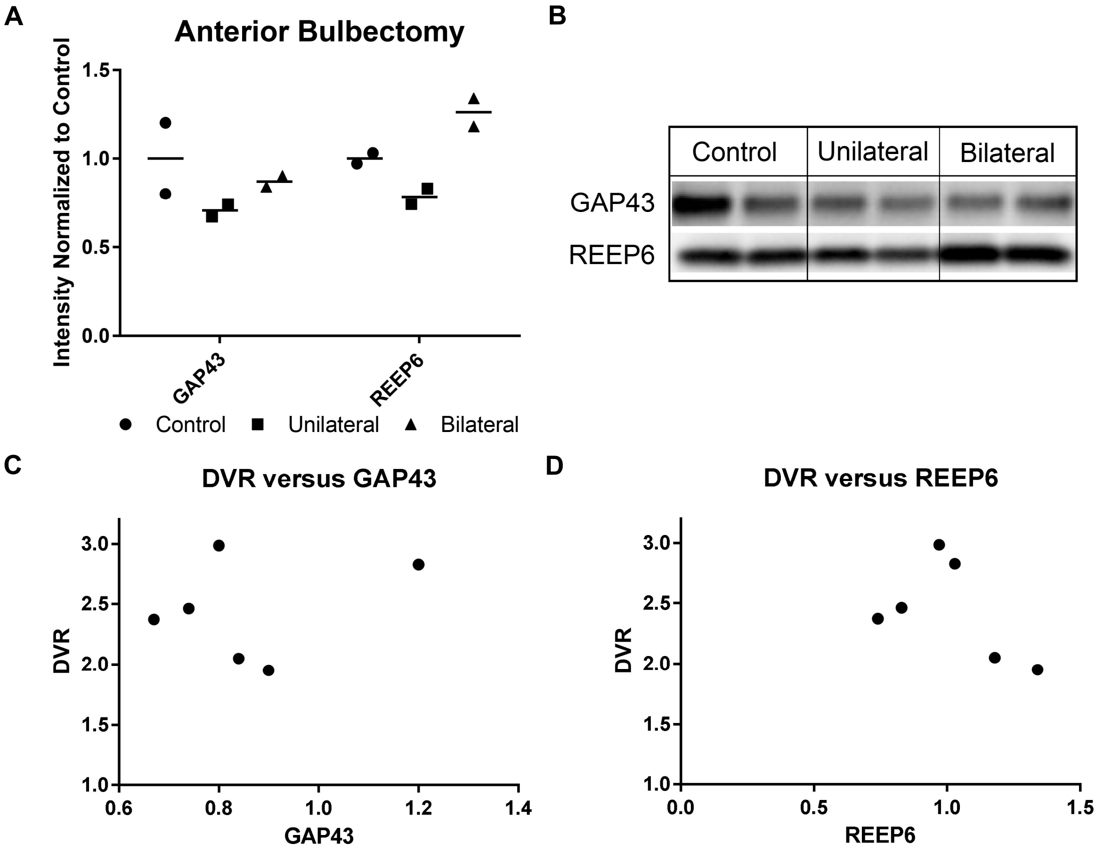

Supplemental Figure 7. Immature OSN and sustentacular cell markers do not correlate with [^{11}C]GV1-57 DVR following anterior bulbectomy. (**A**) Quantification of GAP43 (immature OSNs) and REEP6 (sustentacular cells) immunoreactivity to total OE lysate from control, unilateral bulbectomy and bilateral bulbectomy mice. For each antibody, band intensities were normalized to the average of the control bands. n = 2 per group (control, unilateral, or bilateral) (**B**) Representative images of developed GAP43 and REEP6 immunoblots. (**C**) Scatter plot of individual post-bulbectomy [^{11}C]GV1-57 DVRs (t^* = 45 min) and relative GAP43 immunoreactivities. There is no correlation between [^{11}C]GV1-57 DVR and GAP43 immunoreactivities (Spearman, r = -0.086, P = 0.92). (**D**) Scatter plot of individual post-bulbectomy [^{11}C]GV1-57 DVRs (t^* = 45 min) and relative REEP6 immunoreactivities. There is no correlation between REEP6 immunoreactivities (Spearman, r = -0.49, P = 0.36).

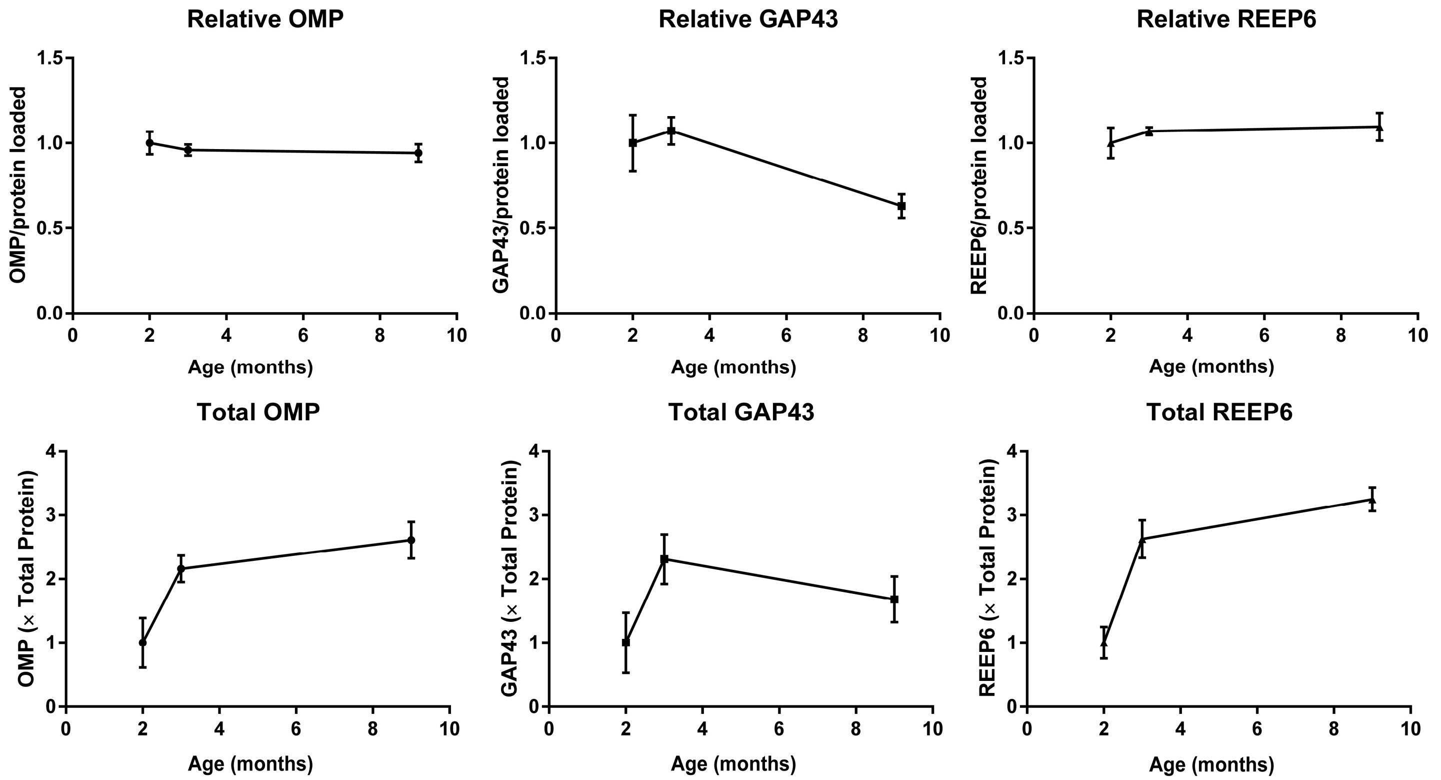

Supplemental Figure 8. Disparate effects of age on mature OSNs, immature OSNs, and sustentacular cells. Immunoblot analysis of mature OSNs (OMP), immature OSNs (GAP43), and sustentacular cells (REEP6) indicates that the mature OSNs and sustentacular cell markers remain at a constant level across development, relative to total protein. Alternatively, the immature OSN marker decreases during development, relative to total OE protein. To achieve a whole-tissue analysis of the cellular markers, the protein levels were multiplied by total protein extracted from septal OE tissue acquired from individual animals. This analysis approximates the whole-tissue, cellular-population analysis obtained from [^{11}C]GV1-57 imaging, and suggests that mature OSNs and sustentacular cells have an increasing cellular population (cellular influx) across the OE developmental period. Immature OSNs have an initially increasing population (cellular influx), which then trends downward between 3 and 9 months of age. This downward trend in the immature OSN population after 3 months of age might indicate an increased maturation rate of immature OSNs in conjunction with decreased rate of new immature OSN production. Error bars are ± SEM; *n* = 3 per age.

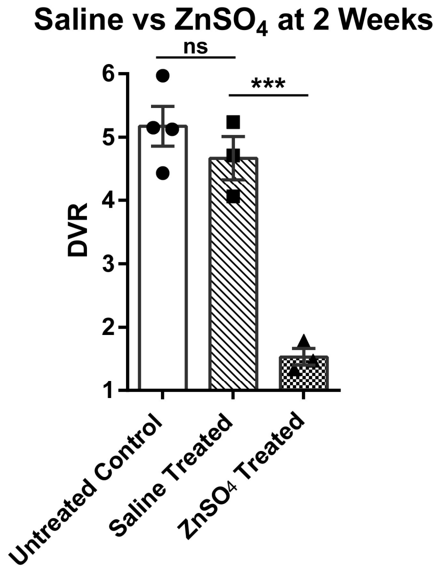

Supplemental Figure 9. Intranasal zinc sulfate significantly reduces [^{11}C]GV1-57 uptake. Quantification of [^{11}C]GV1-57 DVRs (t^* = 45 min) for untreated control rats and rats two-weeks-post intranasal treatment with saline or $ZnSO_4$. Error bars are ± SEM; n = 3–4 per group. ***$P < 0.005$ using a one-tailed Student's t test with a Bonferroni correction ($\alpha = 0.025$) for multiple comparisons; $^{ns}P = 0.33$ using a two-tailed Student's t test with a Bonferroni correction ($\alpha = 0.025$) for multiple comparisons.

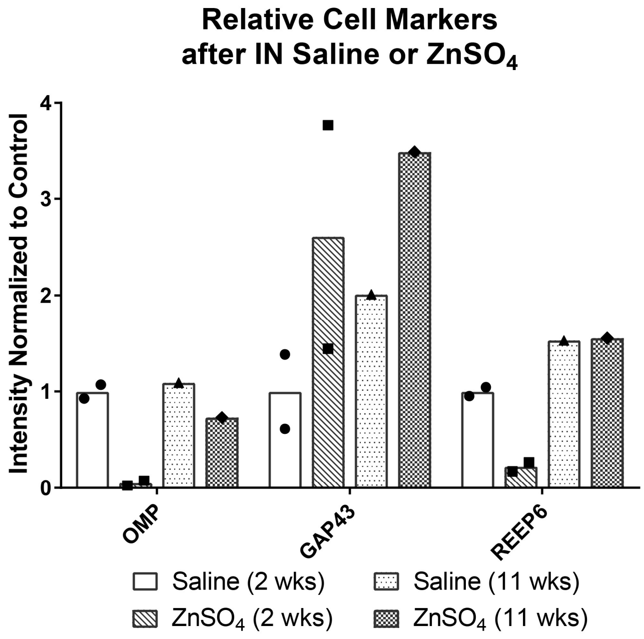

Supplemental Figure 10. Mature OSNs, but not immature OSNs or sustentacular cells, show alterations consistent with [^{11}C]GV1-57 imaging after saline and $ZnSO_4$ treatment. Quantitative analysis of OMP, GAP43, and REEP6 band intensity from OE tissue lysate from rats treated intranasally with saline (100 µl per nostril) or $ZnSO_4$ (10% in saline, 100 µl per nostril) two or eleven weeks prior to OE tissue harvesting. Band intensity normalized to average of saline bands for the respective marker protein. *n* = 1–2 per group.

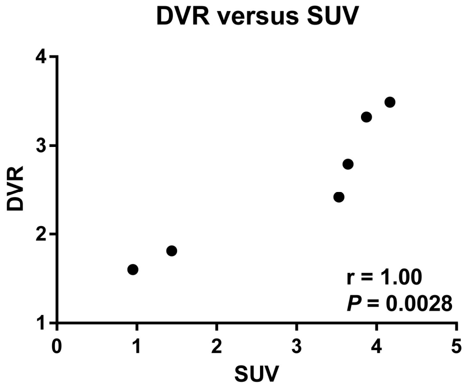

Supplemental Figure 11. Correlation of DVR and SUV analyses of [^{11}C]GV1-57 uptake in the olfactory epithelium. Plot of DVR (t^* = 45 min) versus SUV (3–45 min) shows significant correlation (Spearman, r = 1.00, P = 0.0028) between two quantitative measures of [^{11}C]GV1-57 uptake, indicating radiotracer versatility across quantitative analyses. Graph is comprised of data from a single, longitudinally-imaged, $ZnSO_4$-treated animal.

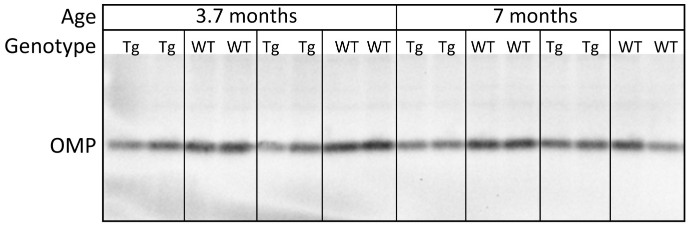

Supplemental Figure 12. Mature OSN population deficit in rTg4510 animals is confirmed by immunoblotting. Raw Western immunoblot data used for quantification of relative mature OSN populations in WT and rTg4510 animals (Figure 5C). For analysis, band intensities were background subtracted, multiplied by total extracted protein per sample, and normalized to the 3.7-month-old WT group. The mature OSN population measured by OMP is significantly ($P < 0.05$) lower in 7-month-old rTg4510 animals compared to age-matched WT controls. Within-age group comparisons were completed with a two-tailed Student's *t* test. $n = 4$ per group.

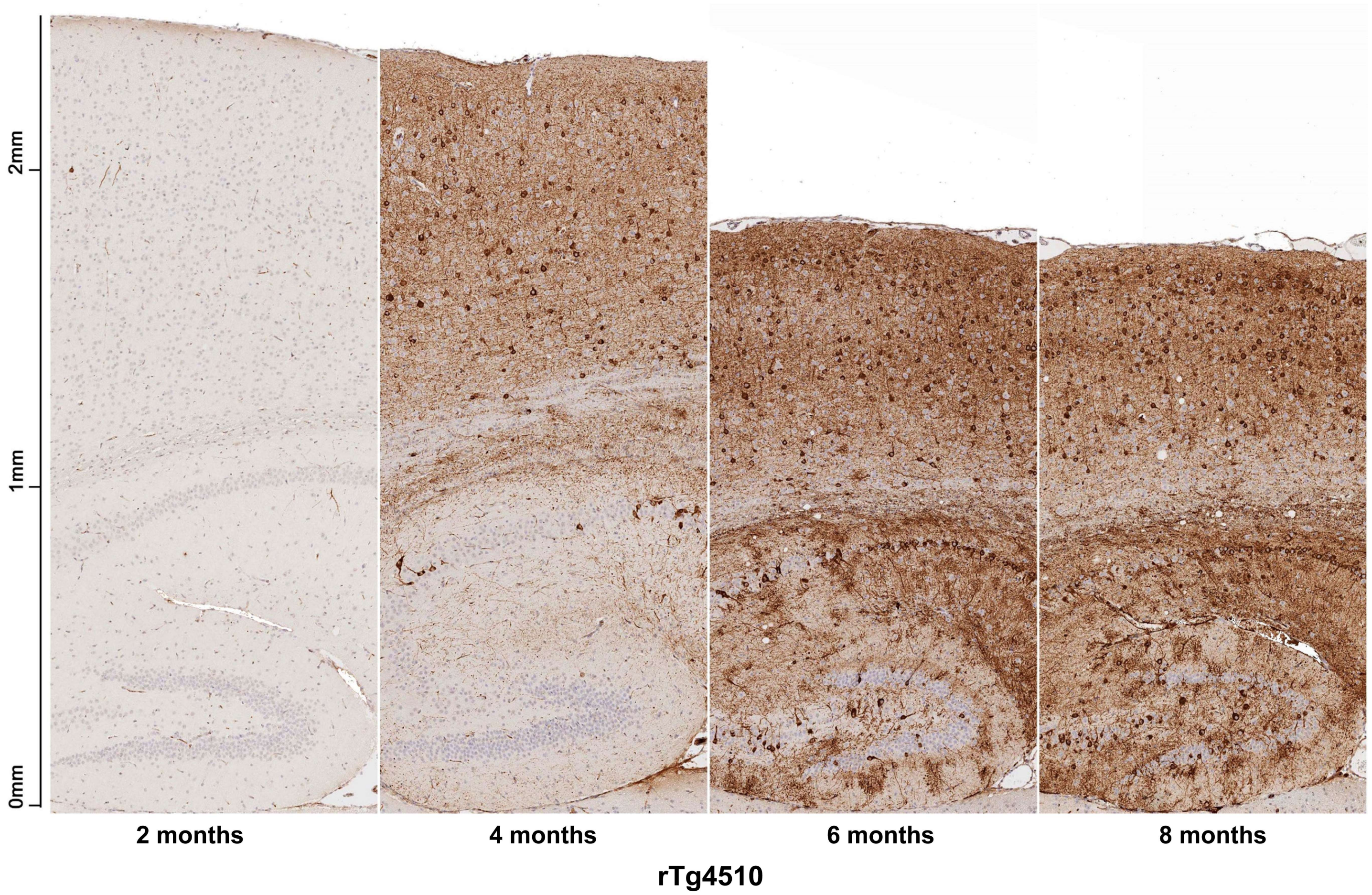

Supplemental Figure 13. Progressive tau-associated neurodegeneration in the rTg4510 mouse model. Cross-sectional analysis of brain tissue from rTg4510 mice shows a progressive increase in tau pathology between 2 and 8 months of age. Neuronal tau accumulation was minimal at 2 months of age and increased substantially by 4 months of age. At 6 months of age, severe cortical and hippocampal tau pathology was associated with significant tissue atrophy and neuronal loss. Tau-associated neurodegeneration was very severe by 8 months of age. Tau pathology was detected immunohistochemically using a phospho-specific tau antibody (PG-5; pSer409) in formalin-fixed paraffin-embedded tissue sections (sagittal plane). Images are representative of their respective time-points; *n* = 15–18 per age.

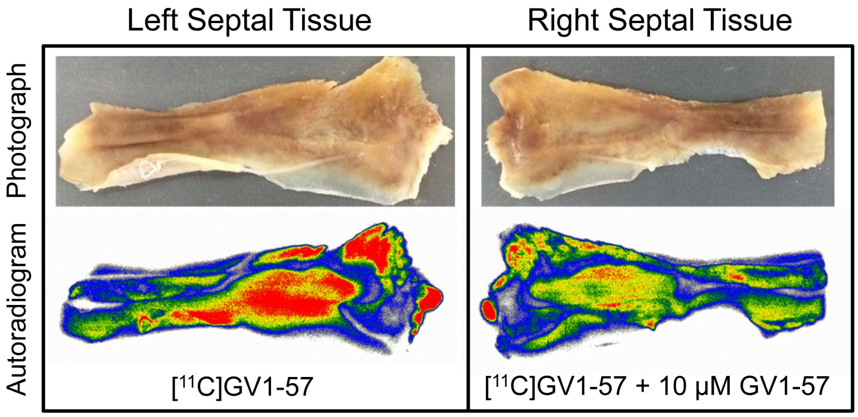

Supplemental Figure 14. [^{11}C]GV1-57 binding in *Papio anubis* nasal tissue. Tissue from either side of a male *Papio anubis* baboon's nasal septum was incubated with either [^{11}C]GV1-57 (left septal tissue) or [^{11}C]GV1-57 following a 20 min incubation with non-radiolabeled GV1-57 (10 μM, right septal tissue). The images on top are photographs of the left and right baboon septal tissue, and the images on the bottom are non-adjusted autoradiograms associated with the left and right septal tissue of the baboon.

Supplemental Table 1

Enzyme Activity Summary:

Kinase:	% Enzyme Activity (relative to DMSO controls) GV1-57 Data 1	Data 2	IC50 (M) Staurosporine*	IC50 (M) Alternate Control cpd*.	Alternate compound ID
**ABL1**	101.84	101.66	**2.76E-08**		
**ABL2/ARG**	95.98	93.43	**1.31E-08**		
**ACK1**	88.97	86.78	**7.05E-08**		
**AKT1**	97.63	94.49	**3.63E-09**		
**AKT2**	104.01	102.87	**1.41E-08**		
**AKT3**	103.23	102.94	**2.54E-09**		
**ALK**	99.44	97.39	**2.05E-09**		
**ALK1/ACVRL1**	110.62	107.35	**ND**	**7.85E-09**	**LDN193189**
**ALK2/ACVR1**	92.41	92.22	**ND**	**2.57E-08**	**LDN193189**
**ALK3/BMPR1A**	97.83	96.04	**ND**	**9.44E-09**	**LDN193189**
**ALK4/ACVR1B**	92.00	90.87	**ND**	**1.65E-07**	**LDN193189**
**ALK5/TGFBR1**	103.27	102.88	**ND**	**1.56E-07**	**LDN193189**
**ALK6/BMPR1B**	109.26	108.56	**ND**	**5.14E-09**	**LDN193189**
**ARAF**	89.95	87.57	**ND**	**1.89E-08**	**GW5074**
**ARK5/NUAK1**	98.17	94.67	**1.08E-09**		
**ASK1/MAP3K5**	90.26	88.46	**6.55E-09**		
**Aurora A**	88.75	88.64	**6.79E-10**		
**Aurora B**	96.98	95.87	**8.81E-09**		
**Aurora C**	95.67	95.00	**1.93E-09**		
**AXL**	102.97	102.61	**6.86E-09**		
**BLK**	92.00	90.17	**9.14E-10**		
**BMPR2**	95.21	95.02	**1.92E-06**		
**BMX/ETK**	100.85	99.88	**4.51E-09**		
**BRAF**	99.68	97.26	**ND**	**1.85E-08**	**GW5074**
**BRK**	84.59	84.34	**2.35E-07**		
**BRSK1**	97.24	96.87	**7.26E-10**		
**BRSK2**	109.54	107.52	**1.95E-09**		
**BTK**	97.17	97.03	**2.10E-08**		
**c-Kit**	110.98	110.25	**9.74E-08**		
**c-MER**	94.38	93.12	**5.92E-09**		
**c-MET**	94.49	93.59	**2.39E-08**		
**c-Src**	96.89	93.62	**1.86E-09**		
**CAMK1a**	100.84	99.14	**2.19E-09**		
**CAMK1b**	83.25	81.59	**3.61E-09**		
**CAMK1d**	92.72	92.70	**1.87E-10**		
**CAMK1g**	99.72	99.16	**4.22E-09**		
**CAMK2a**	95.79	94.94	**2.97E-11**		
**CAMK2b**	94.45	94.21	**3.20E-11**		
**CAMK2d**	101.79	101.59	**4.75E-11**		
**CAMK2g**	94.44	93.56	**3.27E-10**		
**CAMK4**	99.74	98.73	**9.07E-08**		
**CAMKK1**	106.80	103.56	**7.60E-08**		
**CAMKK2**	104.09	101.55	**1.82E-08**		
**CDC7/DBF4**	89.96	89.96	**5.50E-08**		
**CDK1/cyclin A**	93.73	92.56	**2.31E-09**		
**CDK1/cyclin B**	91.40	91.08	**1.34E-09**		
**CDK1/cyclin E**	102.97	99.88	**3.39E-09**		
**CDK14/cyclin Y (PFTK1)**	103.89	103.37	**1.21E-07**		
**CDK16/cyclin Y (PCTAIRE)**	94.06	93.62	**1.54E-08**		

**CDK17/cyclin Y (PCTK2)**	98.30	96.48	**1.32E-08**		
**CDK18/cyclin Y (PCTK3)**	113.73	113.51	**2.96E-08**		
**CDK2/cyclin A**	77.61	76.29	**6.71E-10**		
**CDK2/Cyclin A1**	71.81	71.54	**1.48E-09**		
**CDK2/cyclin E**	72.82	72.39	**1.43E-09**		
**CDK2/cyclin O**	69.00	68.07	**1.18E-09**		
**CDK3/cyclin E**	84.27	82.57	**2.43E-09**		
**CDK4/cyclin D1**	92.31	91.73	**1.83E-08**		
**CDK4/cyclin D3**	84.40	84.10	**4.54E-08**		
**CDK5/p25**	92.84	92.08	**2.25E-09**		
**CDK5/p35**	73.98	73.95	**1.44E-09**		
**CDK6/cyclin D1**	104.43	103.17	**5.89E-09**		
**CDK6/cyclin D3**	90.71	85.98	**9.38E-08**		
**CDK7/cyclin H**	98.88	97.91	**1.33E-07**		
**CDK9/cyclin K**	95.51	94.59	**3.79E-07**		
**CDK9/cyclin T1**	85.43	81.36	**4.39E-09**		
**CHK1**	96.33	94.69	**1.20E-10**		
**CHK2**	88.88	88.08	**5.72E-09**		
**CK1a1**	84.08	82.21	**2.47E-06**		
**CK1a1L**	103.37	101.71	**1.34E-06**		
**CK1d**	66.18	66.03	**ND**	**1.36E-07**	**D4476**
**CK1epsilon**	68.83	68.75	**ND**	**1.56E-07**	**D4476**
**CK1g1**	93.74	93.53	**3.78E-06**		
**CK1g2**	95.74	95.54	**1.52E-06**		
**CK1g3**	88.73	86.91	**1.83E-06**		
**CK2a**	87.38	86.30	**ND**	**7.41E-08**	**GW5074**
**CK2a2**	87.47	85.19	**1.33E-07**		
**CLK1**	102.12	99.35	**1.02E-08**		
**CLK2**	101.21	100.82	**3.57E-09**		
**CLK3**	98.15	95.78	**9.38E-07**		
**CLK4**	106.74	104.29	**9.29E-08**		
**COT1/MAP3K8**	105.42	105.33	**ND**	**1.05E-05**	**Ro-31-8220**
**CSK**	100.62	100.38	**1.32E-08**		
**CTK/MATK**	105.81	102.46	**1.20E-06**		
**DAPK1**	99.48	85.06	**9.56E-09**		
**DAPK2**	103.54	103.12	**2.89E-09**		
**DCAMKL1**	98.46	95.72	**2.09E-07**		
**DCAMKL2**	107.35	107.25	**1.41E-07**		
**DDR1**	102.29	100.97	**3.67E-09**		
**DDR2**	107.60	102.71	**4.44E-10**		
**DLK/MAP3K12**	85.73	85.68	**4.87E-08**		
**DMPK**	100.98	100.83	**1.56E-07**		
**DMPK2**	88.37	86.92	**2.89E-10**		
**DRAK1/STK17A**	93.78	92.66	**2.83E-08**		
**DYRK1/DYRK1A**	93.67	92.44	**2.93E-09**		
**DYRK1B**	85.63	85.11	**7.72E-10**		
**DYRK2**	68.27	68.27	**1.34E-07**		
**DYRK3**	93.40	92.39	**1.09E-08**		
**DYRK4**	99.08	98.80	**ND**	**GW5074**	
**EGFR**	92.82	92.22	**4.71E-08**		
**EPHA1**	91.35	91.01	**8.76E-08**		
**EPHA2**	106.81	105.28	**7.06E-08**		
**EPHA3**	94.83	94.29	**3.51E-08**		
**EPHA4**	95.96	95.84	**1.18E-08**		
**EPHA5**	100.52	97.71	**1.25E-08**		
**EPHA6**	96.99	96.08	**1.93E-08**		
**EPHA7**	91.63	89.47	**2.36E-08**		
**EPHA8**	95.04	91.61	**6.98E-08**		

**EPHB1**	91.60	89.92	**3.21E-08**		
**EPHB2**	96.71	96.05	**4.22E-08**		
**EPHB3**	106.84	105.31	**1.22E-06**		
**EPHB4**	97.09	97.05	**1.42E-07**		
**ERBB2/HER2**	97.23	96.50	**3.17E-08**		
**ERBB4/HER4**	89.20	88.67	**8.88E-08**		
**ERK1**	97.28	96.64	**5.12E-06**		
**ERK2/MAPK1**	82.87	82.22	**3.92E-06**		
**ERK5/MAPK7**	101.09	100.65	**5.12E-06**		
**ERK7/MAPK15**	83.75	82.43	**9.62E-09**		
**ERN1/IRE1**	94.42	92.64	**3.70E-08**		
**ERN2/IRE2**	98.93	98.85	**2.23E-08**		
**FAK/PTK2**	94.94	93.98	**1.44E-08**		
**FER**	111.21	110.50	**1.82E-10**		
**FES/FPS**	94.75	93.24	**1.74E-09**		
**FGFR1**	97.16	97.10	**2.66E-09**		
**FGFR2**	92.52	91.09	**8.57E-10**		
**FGFR3**	113.56	108.07	**6.83E-09**		
**FGFR4**	96.90	96.67	**1.02E-07**		
**FGR**	95.07	92.42	**6.58E-10**		
**FLT1/VEGFR1**	93.15	92.64	**9.69E-09**		
**FLT3**	90.15	90.10	**1.37E-09**		
**FLT4/VEGFR3**	108.79	107.14	**1.32E-09**		
**FMS**	99.93	97.96	**1.92E-09**		
**FRK/PTK5**	107.49	106.55	**5.68E-09**		
**FYN**	103.88	102.69	**1.88E-09**		
**GCK/MAP4K2**	96.28	94.70	**4.73E-10**		
**GLK/MAP4K3**	96.57	95.91	**7.30E-11**		
**GRK1**	98.88	97.83	**7.16E-08**		
**GRK2**	106.69	105.51	**1.21E-06**		
**GRK3**	101.29	100.52	**8.91E-07**		
**GRK4**	91.66	90.95	**6.21E-08**		
**GRK5**	107.91	107.04	**3.04E-08**		
**GRK6**	96.46	95.63	**5.37E-08**		
**GRK7**	98.83	97.54	**5.27E-09**		
**GSK3a**	85.16	83.49	**2.53E-09**		
**GSK3b**	90.05	89.64	**4.86E-09**		
**Haspin**	99.03	97.91	**2.80E-08**		
**HCK**	88.65	85.65	**2.68E-09**		
**HGK/MAP4K4**	89.58	88.57	**2.91E-10**		
**HIPK1**	100.44	98.40	**ND**	**2.31E-07**	**Ro-31-8220**
**HIPK2**	97.92	97.58	**1.39E-07**		
**HIPK3**	90.10	88.78	**2.08E-07**		
**HIPK4**	49.25	48.04	**1.58E-07**		
**HPK1/MAP4K1**	104.54	103.46	**ND**	**2.92E-08**	**Ro-31-8220**
**IGF1R**	96.69	95.73	**2.32E-08**		
**IKKa/CHUK**	98.81	97.06	**1.24E-07**		
**IKKb/IKBKB**	102.23	101.59	**2.15E-07**		
**IKKe/IKBKE**	104.58	102.33	**2.14E-10**		
**IR**	111.29	106.22	**8.36E-09**		
**IRAK1**	98.17	89.86	**1.29E-08**		
**IRAK4**	97.35	82.26	**3.04E-09**		
**IRR/INSRR**	79.80	79.28	**8.35E-09**		
**ITK**	100.76	100.29	**9.85E-09**		
**JAK1**	90.25	88.02	**4.74E-10**		
**JAK2**	94.77	93.95	**1.19E-10**		
**JAK3**	97.19	95.54	**7.89E-11**		
**JNK1**	88.77	87.83	**2.16E-07**		

**JNK2**	97.52	98.05	**8.54E-07**		
**JNK3**	92.26	90.95	**ND**	**5.37E-08**	**JNKi VIII**
**KDR/VEGFR2**	107.60	107.22	**4.12E-09**		
**KHS/MAP4K5**	91.47	90.03	**3.25E-10**		
**KSR1**	103.93	103.68	**6.12E-06**		
**KSR2**	101.32	98.70	**4.08E-06**		
**LATS1**	85.44	84.05	**8.59E-09**		
**LATS2**	92.85	91.88	**1.02E-08**		
**LCK**	99.25	95.84	**2.41E-09**		
**LCK2/ICK**	98.99	98.36	**4.25E-08**		
**LIMK1**	86.43	84.42	**2.70E-09**		
**LIMK2**	103.09	102.12	**2.61E-07**		
**LKB1**	106.13	104.38	**6.86E-08**		
**LOK/STK10**	108.89	108.18	**1.33E-08**		
**LRRK2**	99.09	98.18	**5.69E-09**		
**LYN**	98.08	97.63	**7.36E-10**		
**LYN B**	100.38	98.22	**1.32E-09**		
**MAK**	84.56	83.97	**2.02E-08**		
**MAPKAPK2**	112.41	110.09	**8.14E-08**		
**MAPKAPK3**	97.19	95.40	**2.47E-06**		
**MAPKAPK5/PRAK**	94.54	93.67	**8.52E-08**		
**MARK1**	101.77	101.37	**3.28E-10**		
**MARK2/PAR-1Ba**	95.14	93.54	**1.14E-10**		
**MARK3**	94.61	94.22	**2.60E-10**		
**MARK4**	98.38	98.00	**1.04E-10**		
**MEK1**	99.57	99.19	**1.15E-08**		
**MEK2**	89.56	89.04	**9.41E-09**		
**MEK3**	98.24	96.44	**5.19E-09**		
**MEK5**	100.51	100.10	**1.09E-08**		
**MEKK1**	100.82	99.35	**3.50E-07**		
**MEKK2**	92.54	91.07	**4.34E-08**		
**MEKK3**	86.93	85.87	**3.35E-08**		
**MEKK6**	97.50	96.66	**9.86E-08**		
**MELK**	90.70	89.41	**4.98E-10**		
**MINK/MINK1**	92.87	91.60	**4.22E-10**		
**MKK4**	98.36	98.16	**1.17E-06**		
**MKK6**	96.49	95.54	**2.44E-09**		
**MKK7**	101.78	101.59	**5.11E-07**		
**MLCK/MYLK**	90.48	90.29	**5.58E-08**		
**MLCK2/MYLK2**	93.28	92.21	**9.76E-09**		
**MLK1/MAP3K9**	96.96	96.42	**1.23E-09**		
**MLK2/MAP3K10**	92.21	87.38	**4.37E-09**		
**MLK3/MAP3K11**	95.64	91.45	**6.22E-09**		
**MLK4**	97.54	96.03	**1.15E-06**		
**MNK1**	100.69	100.10	**6.24E-08**		
**MNK2**	98.37	96.06	**1.46E-08**		
**MRCKa/CDC42BPA**	104.10	102.62	**1.60E-09**		
**MRCKb/CDC42BPB**	103.34	103.29	**9.28E-10**		
**MSK1/RPS6KA5**	99.90	97.95	**4.23E-10**		
**MSK2/RPS6KA4**	109.65	108.07	**2.62E-09**		
**MSSK1/STK23**	92.38	91.53	**1.70E-06**		
**MST1/STK4**	99.06	98.74	**1.51E-09**		
**MST2/STK3**	94.01	88.51	**3.78E-09**		
**MST3/STK24**	81.27	77.75	**1.46E-08**		
**MST4**	96.14	94.66	**2.15E-08**		
**MUSK**	79.24	76.20	**6.97E-08**		
**MYLK3**	103.76	102.68	**1.04E-07**		
**MYLK4**	98.94	98.54	**5.24E-08**		

**MYO3A**	95.94	93.10	**1.83E-08**		
**MYO3b**	106.03	101.51	**5.03E-09**		
**NEK1**	100.18	97.42	**8.14E-09**		
**NEK11**	85.18	81.22	**3.65E-07**		
**NEK2**	104.91	100.56	**1.07E-07**		
**NEK3**	110.27	106.95	**3.49E-05**		
**NEK4**	104.66	102.94	**4.93E-08**		
**NEK5**	91.01	88.72	**5.71E-08**		
**NEK6**	100.81	100.33	**ND**	**5.72E-05**	**PKR Inhibitor**
**NEK7**	89.43	86.28	**ND**	**6.79E-06**	**PKR Inhibitor**
**NEK8**	100.73	97.66	**3.65E-08**		
**NEK9**	91.38	89.03	**6.58E-08**		
**NIM1**	98.70	94.10	**7.90E-08**		
**NLK**	76.93	70.93	**5.38E-08**		
**OSR1/OXSR1**	95.43	94.95	**3.97E-08**		
**P38a/MAPK14**	93.56	91.39	**ND**	**1.60E-08**	**SB202190**
**P38b/MAPK11**	101.29	97.95	**ND**	**1.50E-08**	**SB202190**
**P38d/MAPK13**	104.65	102.82	**1.14E-07**		
**P38g**	104.91	100.86	**1.14E-07**		
**p70S6K/RPS6KB1**	98.52	98.46	**5.21E-10**		
**p70S6Kb/RPS6KB2**	98.85	98.63	**6.88E-10**		
**PAK1**	97.03	95.21	**3.91E-10**		
**PAK2**	100.63	98.95	**2.77E-09**		
**PAK3**	94.00	91.82	**3.50E-10**		
**PAK4**	96.35	95.08	**2.37E-08**		
**PAK5**	85.85	85.43	**1.76E-09**		
**PAK6**	98.67	98.17	**5.85E-08**		
**PASK**	98.89	97.74	**6.64E-09**		
**PBK/TOPK**	91.15	89.78	**4.85E-08**		
**PDGFRa**	95.79	95.66	**5.07E-10**		
**PDGFRb**	100.61	100.03	**2.28E-09**		
**PDK1/PDPK1**	81.75	81.33	**5.19E-10**		
**PEAK1**	94.64	94.46	**1.90E-09**		
**PHKg1**	98.44	96.86	**2.57E-09**		
**PHKg2**	98.80	98.41	**5.83E-10**		
**PIM1**	100.39	99.80	**4.27E-09**		
**PIM2**	102.87	102.57	**4.88E-09**		
**PIM3**	98.77	98.49	**9.90E-11**		
**PKA**	100.86	100.85	**5.79E-10**		
**PKAcb**	98.25	97.25	**4.86E-10**		
**PKAcg**	100.50	98.88	**1.84E-09**		
**PKCa**	95.38	94.59	**3.69E-10**		
**PKCb1**	88.33	87.72	**6.79E-09**		
**PKCb2**	94.55	94.19	**2.87E-09**		
**PKCd**	88.90	87.38	**4.28E-10**		
**PKCepsilon**	100.15	96.49	**1.98E-10**		
**PKCeta**	90.89	89.77	**2.42E-10**		
**PKCg**	94.83	93.95	**7.00E-10**		
**PKCiota**	88.86	88.06	**1.34E-08**		
**PKCmu/PRKD1**	90.30	89.17	**8.84E-10**		
**PKCnu/PRKD3**	98.49	98.43	**2.21E-09**		
**PKCtheta**	105.56	99.31	**4.16E-09**		
**PKCzeta**	98.98	95.21	**5.11E-08**		
**PKD2/PRKD2**	116.60	116.41	**9.58E-10**		
**PKG1a**	96.43	95.67	**1.04E-09**		
**PKG1b**	103.68	103.29	**2.57E-09**		
**PKG2/PRKG2**	101.05	98.30	**8.84E-09**		
**PKN1/PRK1**	114.33	105.42	**2.66E-09**		

PKN2/PRK2	107.59	104.77	1.46E-08		
PKN3/PRK3	102.66	98.53	8.48E-09		
PLK1	99.42	99.22	1.23E-07		
PLK2	97.93	97.90	2.41E-07		
PLK3	104.32	104.21	1.43E-07		
PLK4/SAK	97.33	96.74	9.20E-09		
PRKX	103.85	101.25	1.81E-09		
PYK2	101.09	100.97	9.74E-09		
RAF1	94.91	94.29	ND	9.04E-09	GW5074
RET	98.47	97.81	2.36E-09		
RIPK2	114.97	112.90	3.89E-07		
RIPK3	89.08	86.79	ND	2.85E-06	GW5074
RIPK5	97.76	94.99	4.71E-08		
ROCK1	99.76	99.74	4.53E-10		
ROCK2	103.11	102.97	4.99E-10		
RON/MST1R	99.10	96.20	7.96E-08		
ROS/ROS1	85.79	84.08	1.23E-10		
RSK1	96.54	95.19	9.02E-11		
RSK2	97.41	96.86	9.48E-11		
RSK3	104.60	103.72	1.37E-10		
RSK4	99.52	99.40	1.06E-10		
SBK1	96.76	96.62	2.24E-07		
SGK1	87.74	87.30	5.73E-09		
SGK2	90.62	88.14	1.15E-08		
SGK3/SGKL	101.94	100.65	7.90E-08		
SIK1	84.76	83.64	4.40E-10		
SIK2	95.18	93.76	3.25E-10		
SIK3	94.88	94.20	6.46E-10		
SLK/STK2	94.86	94.34	1.59E-08		
SNARK/NUAK2	95.73	94.73	1.58E-09		
SNRK	105.40	104.61	5.43E-09		
SRMS	90.83	90.20	2.39E-06		
SRPK1	93.09	91.89	2.47E-08		
SRPK2	95.79	94.25	1.79E-07		
SSTK/TSSK6	102.77	100.26	1.65E-07		
STK16	98.82	98.04	2.66E-07		
STK21/CIT	101.85	99.78	4.33E-07		
STK22D/TSSK1	68.59	67.59	4.42E-11		
STK25/YSK1	100.76	97.51	4.98E-09		
STK32B/YANK2	103.44	103.35	3.66E-08		
STK32C/YANK3	94.97	90.26	1.46E-07		
STK33	92.50	90.98	3.68E-08		
STK38/NDR1	98.16	97.74	5.23E-09		
STK38L/NDR2	92.48	92.13	1.11E-09		
STK39/STLK3	100.15	99.74	2.63E-08		
SYK	94.29	94.28	2.56E-10		
TAK1	100.99	98.96	8.82E-08		
TAOK1	107.69	105.48	3.99E-10		
TAOK2/TAO1	97.30	96.50	4.21E-09		
TAOK3/JIK	106.95	106.75	2.13E-09		
TBK1	103.78	101.91	2.16E-09		
TEC	102.71	101.32	2.48E-08		
TESK1	97.38	96.75	1.63E-07		
TGFBR2	107.82	104.59	ND	1.36E-07	LDN193189
TIE2/TEK	93.01	91.93	2.52E-08		
TLK1	98.48	97.86	2.75E-08		
TLK2	107.14	106.31	3.08E-09		
TNIK	98.73	98.26	8.09E-10		

**TNK1**	104.02	103.17	**2.03E-09**		
**TRKA**	96.62	96.40	**1.18E-09**		
**TRKB**	100.87	100.41	**8.02E-11**		
**TRKC**	85.55	85.25	**1.92E-10**		
**TSSK2**	95.50	94.66	**2.43E-09**		
**TSSK3/STK22C**	95.60	92.34	**3.06E-09**		
**TTBK1**	97.21	92.86	**ND**	**1.80E-05**	**SB202190**
**TTBK2**	92.08	89.86	**ND**	**5.07E-06**	**SB202190**
**TXK**	96.63	96.42	**2.49E-08**		
**TYK1/LTK**	100.28	98.89	**3.00E-08**		
**TYK2**	94.84	94.22	**1.54E-10**		
**TYRO3/SKY**	96.57	93.50	**1.85E-09**		
**ULK1**	118.66	118.46	**1.94E-08**		
**ULK2**	102.24	101.75	**2.31E-09**		
**ULK3**	97.28	96.12	**2.61E-09**		
**VRK1**	90.52	89.75	**ND**	**2.15E-07**	**Ro-31-8220**
**VRK2**	106.18	105.57	**ND**	**2.43E-05**	**Ro-31-8220**
**WEE1**	103.72	100.08	**ND**	**2.33E-06**	**Wee-1 Inhibitor**
**WNK1**	82.94	71.49	**1.07E-05**		
**WNK2**	89.93	86.71	**1.25E-06**		
**WNK3**	69.68	68.06	**ND**	**1.44E-06**	**Wee-1 Inhibitor**
**YES/YES1**	101.12	100.69	**1.96E-09**		
**YSK4/MAP3K19**	103.94	103.38	**5.37E-09**		
**ZAK/MLTK**	90.02	85.16	**ND**	**8.43E-07**	**GW5074**
**ZAP70**	110.24	108.05	**3.22E-09**		
**ZIPK/DAPK3**	97.23	95.79	**2.38E-09**		

*** Empty cells indicate no inhibition or compound activity that could not be fit to an IC50 curve**

ND **Indicates compound not tested against enzyme**
